# Associations of beans, bean products, and soy isoflavone intake with chronic kidney disease: a cross-sectional study

**DOI:** 10.1186/s12937-026-01380-0

**Published:** 2026-08-05

**Authors:** Song Zhang, Chao Ji, Yang Xia, Yuhong Zhao

**Affiliations:** 1 Department of Clinical Epidemiology, Shengjing Hospital of China Medical University, No. 36, San Hao Street, Shenyang, Liaoning Province 110004 China; 2 School of Public Health, China Medical University, Shenyang, China; 3 Liaoning Key Laboratory of Precision Medical Research On Major Chronic Disease, Shenyang, China

**Keywords:** Bean, Bean product, Soy isoflavones, CKD, Cross-sectional study

## Abstract

**Background:**

Current evidence on the association between beans and bean products intake and chronic kidney disease (CKD) remains limited. In vitro and animal studies suggest that soy isoflavones exhibit antioxidant and anti-inflammatory properties, potentially conferring renal protective effects. However, large-scale epidemiological studies are lacking to clarify the association between soy isoflavone intake and CKD.

**Objective:**

Investigating the association between beans and bean products intake, soy isoflavones, and CKD in the Northeast China Biobank (NEC-Biobank) based on Northeast China Regional Population.

**Methods:**

From July 2018 to December 2019, we enrolled 27,003 participants aged ≥ 18 years. Dietary intake was assessed using a validated 110-item semi-quantitative Food Frequency Questionnaire (FFQ). CKD was defined by reduced estimated glomerular filtration rate (eGFR) or the presence of proteinuria. The intake of each independent variable was adjusted using the nutrient residual method. Multivariable logistic regression models were employed to examine associations between beans and bean products residual-adjusted intake, soy isoflavones, and CKD prevalence. Restricted cubic spline (RCS) analyses were performed to evaluate potential nonlinear associations.

**Results:**

Among 27,003 participants, 900 individuals had CKD. After multivariable adjustment using the lowest intake quartile as reference, we observed residual-adjusted intake the highest level of total beans and bean products was inversely associated with the prevalence of CKD (Odds Ratios [OR] and 95% Confidence Intervals [CI]) were 0.67, (0.54–0.82), *P *_*for trend*_ < 0.001. Similar results were also observed in the association between soy isoflavones and CKD. Compare with the lowest quartile, OR and 95% CI of highest intake of soy isoflavones were 0.70 (0.58–0.86), *P *_*for trend*_ < 0.001. In subgroup analysis, the association was also observed in all other subgroups except for the Han ethnicity group.

**Conclusion:**

Intake of beans/bean products and soy isoflavones was inversely associated with CKD prevalence, indicating a potential protective relationship. However, due to the cross-sectional design, these observational findings cannot establish causality and warrant confirmation in future large-scale prospective studies.

**Supplementary Information:**

The online version contains supplementary material available at https://doi.org/10.1186/s12937-026-01380-0.

## Introduction

Chronic kidney disease (CKD) is defined as a progressive condition characterized by sustained kidney dysfunction for more than three months, diagnosed when the estimated glomerular filtration rate (eGFR) falls below 60 mL/(min·1.73m^2^) or when proteinuria is present [1]. This irreversible deterioration of renal function may ultimately lead to end-stage renal disease (ESRD) [2]. As a fatal progressive illness, CKD affects over 10% of the global population and ranks among the leading causes of mortality worldwide [3]. In 2019, the disease accounted for 18,986,903 cases and 1,427,232 deaths globally, with age-standardized incidence rates, prevalence, mortality, and disability-adjusted life years (DALYs) consistently rising between 1990 and 2019 [4]. Projections indicate that by 2040, CKD will become the fifth most common cause of death globally [5].

The clinical and socioeconomic burdens of CKD are substantial. Patients typically require lifelong renal replacement therapy (RRT) [6]. CKD is not only a direct cause of death but also a risk amplifier for other chronic non-communicable diseases [7]. In China, the burden of CKD is substantial, with an estimated 82 million cases in 2019 and higher age-standardized rates observed among women and individuals aged ≥ 70 years. Moreover, age-standardized CKD rates have steadily increased from 1990 to 2019 [8]. Rapid economic development and population aging further exacerbate this burden. Annual treatment expenditures exceed tens of billions, with patients with ESRD incurring disproportionately high hospitalization costs [9]. These trends underscore the urgent need for effective prevention strategies.

Beans and bean products have long been recognized as important sources of high-quality protein within traditional diets [10]. These nutrient-dense foods contain abundant proteins, carbohydrate complexes, fatty acids, and various bioactive phytochemicals [11]. Current epidemiological evidence indicates that bean consumption provides protective effects against several chronic diseases, including hypertension, diabetes, and various cancers, thereby supporting global dietary recommendations to increase bean intake [12]. A rural Chinese cohort study reported significant associations between bean consumption and reduced CKD prevalence [13]. Mechanistic studies further indicated that bean-derived bioactive components would improve renal hemodynamics, reduce proteinuria, and mitigate histological kidney damage, thereby potentially slowing disease progression [14].

Among the bioactive constituents, soy isoflavones as the primary functional compounds in beans exhibit potent antioxidant, anti-inflammatory, and anticarcinogenic properties [15]. The naturally occurring soy isoflavones in beans are divided into three categories: daidzein, glycitein, and genistein [16]. Structurally analogous to human 17-β estradiol, these compounds exert biological effects by activating estrogen receptors to mimic endogenous hormone activity [17]. Emerging evidence from animal and in vitro studies suggests isoflavone-rich diets may attenuate the development of renal dysfunction [18]. However, the small sample sizes in existing studies limit the ability to draw definitive conclusions about their protective effects against CKD [19].

To date, research examining the association between the intake of beans and bean products and CKD has been limited to rural Chinese populations, and the relationship between soy isoflavone consumption and CKD remains unclear. Therefore, we investigated whether the consumption of beans and their products, as well as soy isoflavones, is associated with a lower prevalence of CKD.

## Methods

### Study population

**Fig. 1:**
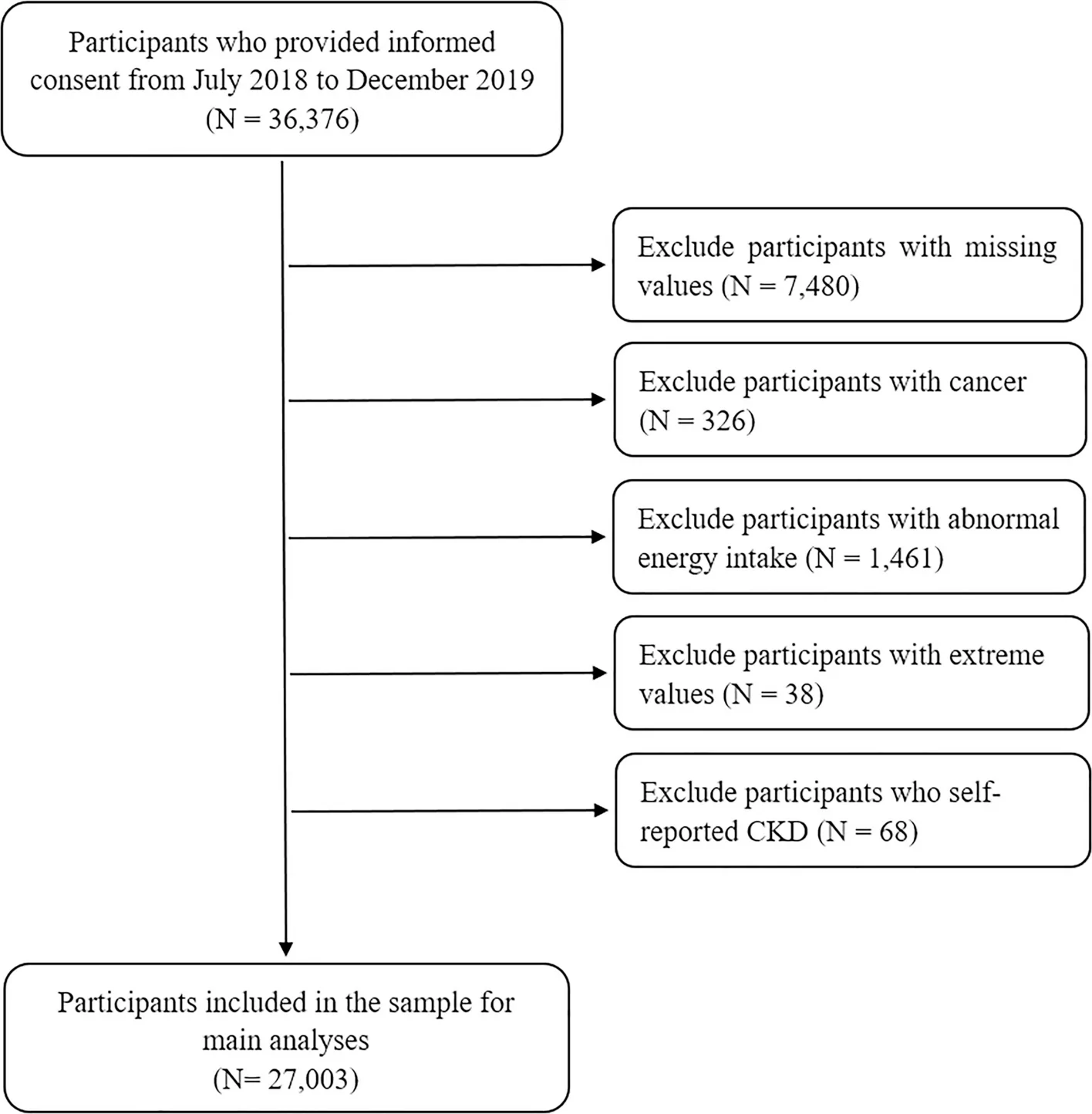
Inclusion and exclusion figure of the study population from the Northeast Natural Population Cohort

This cross-sectional study was based on the Northeast China Biobank (NEC-biobank) study, a large prospective cohort study of a population living in four provinces in Northeast China [20]. The study involved multiple components including questionnaire surveys, physical examinations, biological sample processing, and laboratory tests. The NEC-biobank study was launched in July 2018, and a total of 36,376 participants were recruited. After excluding individuals with missing data (*n* = 7,480; Supplementary Table 1), cancer patients (*n* = 326) [21], those with abnormal energy intake (*n* = 1,461; Male: > 6000 kcal or < 800 kcal; Female: > 4000 kcal or < 600 kcal) [22], outliers (*n* = 38; > 3 standard deviations from the mean), and those who self-reported CKD (*n* = 68). Because individuals with self-reported CKD may have intentionally modified their diet after diagnosis, they were excluded to reduce potential reverse causation bias. After these exclusions, 27,003 participants were ultimately included in this analysis (Fig. 1). All participants provided written informed consent. Throughout the study, all participants were conducted in compliance with ethical standards and approved by the Ethics Committee of Shengjing Hospital of China Medical University (No. 2017PS190K).

### Dietary assessment

A semi-quantitative Food Frequency Questionnaire (FFQ) was used to assess the frequency of consumption of specific foods over a defined period. The 110 food items included in the FFQ were adapted from established national and project-specific FFQs and were further refined to reflect regional dietary patterns in Northeast China as well as expert recommendations. The reliability and validity of the FFQ have been demonstrated in previous studies [23]. The items of beans and bean products were categorized as follows: beans included soybeans, mung beans, various snap beans, bean sprouts and other beans; bean products included soy milk, soybean milk, tofu pudding, tofu, dried tofu sticks and dried tofu. Consumption frequency was classified into seven categories: ≥ 2 times/day; once/day; 4–6 times/week; 2–3 times/week; once/week; 2–3 times/month; and rarely/never. Participants reported their frequency of consumption for each food and beverage over the previous year.

The consumption of each food item was calculated by multiplying the daily consumption frequency by the portion size (in grams) to obtain the estimated daily intake. Nutrient and energy intake were computed using data from the Chinese Food Composition Table (Sixth Edition) [24]. To control for the confounding effect of energy intake, the nutrient residual method was used to adjust the intakes of beans and bean products, as well as soy isoflavones and their subtypes, thereby isolating the effects of these variables independent of total energy. For primary analyses, Total beans and bean products residual-adjusted intake was categorized into quartiles (Q1-Q4): Q1 (< 44.77 g/day), Q2 (44.77–68.47 g/day), Q3 (68.48–100.91 g/day), and Q4 (≥ 100.92 g/day). Similarly, soy isoflavones residual-adjusted intake was grouped as Q1 (< 8.68 mg/day), Q2 (8.68–13.92 mg/day), Q3 (13.93–21.32 mg/day), and Q4 (≥ 21.33 mg/day). To assess the potential confounding effects of dietary patterns on the association between beans/bean products residual-adjusted intake and soy isoflavone residual-adjusted intake with CKD [25], this study employed exploratory factor analysis (EFA) to extract dietary patterns. EFA reduces the dimensionality of overall food intake information into several major factors based on correlations between food intakes. The 110 food items were categorized into the 21 dietary groups (excluding beans and bean products) [26]. Factor analysis was conducted to yield dietary patterns and factor loadings for all 21 food groups in grams. Varimax rotation was applied to increase data interpretability. Finally, five major dietary patterns were identified using a scree plot, and scores for each dietary pattern were calculated and included as covariates in the primary analysis [27].

### Specimen collection

Participants underwent an overnight fast from food and water for at least 8–10 h, and fasting antecubital venous blood samples were collected in the early morning. Blood specimens were obtained using EDTA anticoagulant tubes and serum separator tubes containing a clot activator with separating gel. All samples were transported to the laboratory and tested within 4 h after collection, with assays including blood glucose, serum creatinine, and other parameters. Urine specimens were collected as either first-morning void or random urine; female participants were required to avoid menstruation. Urine samples were placed in screw-capped centrifuge tubes and also sent for analysis within 4 h, with measurements covering urinary albumin, urinary occult blood, and red blood cell counts under high-power field microscopy, among other items. All samples were collected once, and the above indicators were measured once, with abnormal results repeated according to the retest criteria. All analyses were performed using the Sysmex XE-2100 automated hematology analyzer system and the HX-AU5800 automated biochemistry analyzer. Proteinuria was detected using urine dipsticks, and if it is found to be positive, it is judged to have appeared Proteinuria.

### Diagnosis of CKD

Participants were considered to have CKD if they met at least one of the following criteria: (a) the presence of proteinuria, defined as a positive urine protein test [28]; (b) an estimate glomerular filtration rate (eGFR) < 60 mL/min/1.73m^2^, calculated using the 2021 CKD-EPI creatinine equation [29].

### Measurement of covariates

The assessment of covariates was conducted using standardized questionnaires to collect demographic characteristics, including age, sex, ethnicity, smoking and alcohol consumption status, annual family income, and education level. Total physical activity levels were calculated based on the physical activity scale from the China Kadoorie Biobank study [30], measured in metabolic equivalent hours per week (MET-h/wk). Anthropometric parameters, including height, weight, and waist circumference (obesity defined as > 90 cm for men and > 85 cm for women), were measured by trained investigators using standardized equipment. Body mass index (BMI) was calculated as weight in kilograms divided by height in meters squared. Blood pressure was measured using an Omron HBP-1300 electronic sphygmomanometer in an independent and quiet environment, with three measurements taken at one-minute intervals, recorded to the nearest 1 mmHg. Hypertension was diagnosed based on self-reported disease history in the questionnaire or systolic blood pressure > 140 mmHg and/or diastolic blood pressure > 90 mmHg [31]. Diabetes diagnosis was based on self-reported disease history or fasting blood glucose ≥ 7 mmol/L [32]. Hyperuricemia was defined as serum uric acid > 420 μmol/L [33].

### Statistical analysis

After normality testing indicated that continuous variables did not follow a normal distribution, the Wilcoxon test or the Kruskal–Wallis test was used to calculate *P*-values, with variables described as medians (interquartile ranges). Categorical variables were analyzed using chi-square tests, presented as counts (percentages). Multivariable logistic regression models examined associations between these exposures and CKD, treating beans, bean products, and soy isoflavone residual-adjusted intake as independent variables (with Q1 as reference) and CKD status as the dependent variable. Three statistical models were fitted: (1) Crude model without adjustments; (2) Model 2: Adjusted for age, sex, ethnicity, BMI, hypertension, diabetes, smoking, alcohol consumption, annual family income, education level, physical activity, and energy intake; (3) Model 3: Further adjusted for dietary patterns (without beans and bean products) on top of Model 2. Variance inflation factors (VIF) assessed multicollinearity in the final model [34], showing no severe collinearity (VIF range: 1.06–6.33). *P *_*for trend*_ was calculated by coding the median residual-adjusted intake values of each quartile as continuous variables. Stratified analyses by sex, age (< 50 and ≥ 50 years), ethnicity, BMI (< 24 and ≥ 24 kg/m^2^), hypertension (yes and no), and diabetes (yes and no) were conducted, with likelihood ratio tests evaluating multiplicative interactions. To explore the dose-dependent association with prevalence of CKD, beans and bean products residual-adjusted intake, soy isoflavones were treated as a continuous variable (per SD increment) in the analyses. Restricted cubic splines (RCS) with three knots (10th, 50th, 90th percentiles) assessed nonlinear associations between residual-adjusted intake and CKD [35]. The median of the residual-adjusted intake was used as the reference value in the RCS models.

Sensitivity analyses included: (1) Excluded individuals under the age of 40, as CKD under 40 is more likely to be related to genetic factors rather than acquired poor dietary habits; (2) Excluding those with recent dietary changes (during the previous year); (3) Incorporating the prevalence of hyperuricemia into covariates in the model 3 to adjust the regression model [36]; (4) The K/DOQI guidelines developed by the National Kidney Foundation were used as the diagnostic basis for CKD to observe whether the results were robust. (5) Protein, sodium, and red meat intake were adjusted for by including them in multivariate regression models. (6) Using urine protein positivity as the outcome, excluding participants with isolated reduced eGFR. (7) Using reduced eGFR as the outcome, excluding participants with isolated urine protein positivity.

All analyses were performed using SAS (9.4; SAS Institute Inc.) and R (4.6.0), with two-sided *P*-values < 0.05 considered statistically significant. To account for the inflated Type I error rate from multiple testing in the subgroup and interaction analyses, we used the Bonferroni correction. For each subgroup analysis, the two-sided significance level (α = 0.05) was divided by the number of tests (*n* = 3), which included the trend tests for both strata and the interaction test, yielding a corrected statistical significance threshold of *P* < 0.0167.

## Results

### Characteristics of participants

The baseline characteristics of participants, stratified by quartiles of soy isoflavone residual-adjusted intake, are presented in Table 1. This study included a total of 27,003 participants, of whom 900 (3.33%) had CKD. Of the total participants, 808 (2.99%) had proteinuria, 157 (0.58%) had reduced eGFR, and 65 (0.24%) had both reduced eGFR and proteinuria. The majority of participants were over 49 years old, with 17,531 (64.92%) being women. Compared with participants in the lowest isoflavone residual-adjusted intake group, those in the highest group were older and included a higher proportion of women and individuals of other ethnicity. They also had lower educational attainment and lower annual household income. In addition, the highest isoflavone residual-adjusted intake group showed lower rates of smoking and drinking and had higher fasting blood glucose, lower serum uric acid levels, and a greater prevalence of chronic conditions (hypertension and diabetes). Compared with the group with the lowest residual-adjusted intake, the group with the highest residual-adjusted intake had lower total energy and carbohydrate intake, but higher fat and protein intake. In terms of dietary intake, individuals with the highest isoflavone residual-adjusted intake consumed less meat but more fish and vegetables than those with the lowest intake. Additionally, the prevalence of CKD in the highest residual-adjusted intake group was lower (all *P* < 0.05). The baseline characteristics for residual-adjusted intake stratification based on the beans and bean products is basically consistent with the above (Supplementary Table 2).

**Table 1:** Baseline characteristics of study participants stratified by Soy isoflavones residual-adjusted intake

Variables	Total (*N* = 27,003)	Quartile of Soy isoflavones residual-adjusted intake				*P*-value
		Q1 (n = 6750)	Q2 (*n* = 6751)	Q3 (*n* = 6751)	Q4 (*n* = 6751)	
Age, years, n (%)						< 0001
< 50	9296 (34.43)	2633 (39.01)	2306 (34.16)	2104 (31.17)	2253 (33.37)	
≥ 50	17,707 (65.57)	4117 (60.99)	4445 (65.84)	4647 (68.83)	4498 (66.63)	
Sex, n (%)						< 0001
Men	9472 (35.08)	3236 (47.94)	2059 (30.50)	1857 (27.51)	2320 (34.37)	
Women	17,531 (64.92)	3514 (52.06)	4692 (69.50)	4894 (72.49)	4431 (65.63)	
Ethnicity, n (%)						< 0001
Han	14,581 (54.00)	3239 (47.99)	3953 (58.55)	4125 (61.10)	3264 (48.35)	
Other	12,422 (46.00)	3511 (52.01)	2798 (41.45)	2626 (38.90)	3487 (51.65)	
Education, n (%)						< 0001
Middle school and below	16,188 (59.95)	3604 (53.39)	4215 (62.44)	4435 (65.69)	3934 (58.27)	
High school	5452 (20.19)	1483 (21.97)	1252 (18.55)	1284 (19.02)	1433 (21.23)	
College and above	5363 (19.86)	1663 (24.64)	1284 (19.02)	1032 (15.29)	1384 (20.50)	
Annual family income, RMB, n (%)						< 0001
< 50,000	11,522 (42.67)	2657 (39.36)	2936 (43.49)	3176 (47.04)	2753 (40.78)	
50,000–100,000	10,983 (40.67)	2680 (39.70)	2713 (40.19)	2627 (38.91)	2963 (43.89)	
> 100,000	4498 (16.66)	1413 (20.93)	1102 (16.32)	948 (14.04)	1035 (15.33)	
Current smoker, n (%)	5862 (21.71)	1993 (29.53)	1319 (19.54)	1166 (17.27)	1384 (20.50)	< 0001
Current drinker, n (%)	6073 (22.49)	2346 (34.76)	1272 (18.84)	1051 (15.57)	1404 (20.80)	< 0001
Physical activity, MET-h/wk, n (%)						< 0001
≤ 50	7379 (27.33)	1696 (25.13)	1987 (29.43)	2053 (30.41)	1643 (24.34)	
51–100	8405 (31.13)	1972 (29.21)	2098 (31.08)	2127 (31.51)	2208 (32.71)	
101–150	4816 (17.83)	1337 (19.81)	1151 (17.05)	1098 (16.26)	1230 (18.22)	
> 150	6403 (23.71)	1745 (25.85)	1515 (22.44)	1473 (21.82)	1670 (24.74)	
BMI, kg/m^2^, n (%)						0.297
< 24	9969 (36.92)	2481 (36.76)	2532 (37.51)	2435 (36.07)	2521 (37.34)	
≥ 24	17,034 (63.08)	4269 (63.24)	4219 (62.49)	4316 (63.93)	4230 (62.66)	
Waist, n (%)						0.928
Normal	12,862 (47.63)	3215 (47.63)	3234 (47.90)	3218 (47.67)	3195 (47.33)	
Obesity	14,141 (52.37)	3535 (52.37)	3517 (52.10)	3533 (52.33)	3556 (52.67)	
SBP, mmHg, median, (IQR)	131.34 (119.67, 145.67)	129.67 (118.33, 142.67)	131.00 (119.00, 145.67)	132.67 (120.67, 148.00)	131.67 (120.00, 146.34)	< 0001
DBP, mmHg, median, (IQR)	79.34 (72.00, 87.00)	79.00 (71.67, 86.67)	79.34 (72.00, 87.00)	80.00 (72.34, 87.34)	79.00 (71.67, 86.67)	< 0001
FBG, mmol/L, median, (IQR)	5.33 (4.91, 5.90)	5.29 (4.85, 5.82)	5.30 (4.90, 5.84)	5.35 (4.97, 5.90)	5.40 (4.98, 6.00)	< 0001
SUA, μmol/L, median, (IQR)	302.00 (252.70, 364.20)	313.80 (260.50, 379.50)	298.60 (251.20, 357.60)	296.60 (250.40, 353.60)	301.30 (250.20, 365.50)	< 0001
Hypertension, n (%)	11,087 (41.06)	2595 (38.44)	2726 (40.38)	2949 (43.68)	2817 (41.73)	< 0001
Diabetes, n (%)	3146 (11.65)	666 (9.87)	790 (10.81)	833 (12.34)	917 (13.58)	< 0001
Hyperuricemia, n (%)	3120 (11.55)	1004 (14.87)	690 (10.22)	628 (9.30)	798 (11.82)	< 0001
Energy intake, kcal/d, median, (IQR)	1371.09 (1045.71, 1796.07)	1694.26 (1417.89, 2037.52)	1199.40 (1000.79, 1425.07)	1035.59 (846.21, 1497.05)	1529.38 (1215.39, 1957.50)	< 0001
Carbohydrate intake, g/d, median, (IQR)	209.81 (163.25, 269.46)	259.01 (216.43, 311.42)	192.19 (159.00, 227.39)	166.23 (133.47, 224.55)	220.66 (176.29, 279.87)	< 0001
Protein intake, g/d, median, (IQR)	55.28 (42.00, 73.55)	63.86 (52.20, 78.25)	47.02 (38.34, 57.24)	43.38 (35.17, 61.49)	68.08 (53.61, 87.44)	< 0001
Fat intake, g/d, median, (IQR)	32.92 (22.89, 46.88)	40.45 (30.00, 53.25)	26.83 (19.90, 35.74)	24.84 (18.30, 37.72)	41.13 (30.23, 55.01)	< 0001
Meat intake, g/d, median, (IQR)	42.84 (17.16, 64.92)	60.00 (28.56, 85.56)	42.84 (13.50, 64.26)	30.00 (13.50, 60.00)	42.84 (17.16, 64.26)	<.0001
Fish intake, g/d, median, (IQR)	20.45 (9.84, 31.69)	19.68 (8.20, 32.13)	18.86 (8.20, 57.69)	20.02 (9.84, 31.69)	21.32 (13.94, 35.01)	<.0001
Vegetable intake, g/d, median, (IQR)	193.14 (123.57, 291.65)	219.18 (142.46, 313.96)	161.23 (109.50, 246.00)	163.31 (107.57, 251.06)	238.01 (156.64, 339.11)	<.0001
Fruit intake, g/d, median, (IQR)	115.62 (49.86, 224.90)	153.13 (69.60, 280.08)	100.95 (43.46, 192.78)	81.19 (29.11, 167.83)	140.25 (64.26, 254.73)	<.0001
CKD, n (%)	900 (3.33)	286 (4.24)	222 (3.29)	201 (2.98)	191 (2.83)	<.0001
Urine protein, n (%)	808 (2.99)	261 (3.87)	199 (2.95)	178 (2.64)	170 (2.52)	<.0001
eGFR < 60 mL/min/1.73 m^2^, n (%)	157 (0.58)	52 (0.77)	44 (0.65)	32 (0.47)	29 (0.43)	0.032
Both, n (%)	65 (0.24)	27 (0.40)	21 (0.31)	9 (0.13)	8 (0.12)	0.001

Since none of the continuous variables followed a normal distribution, the Kruskal–Wallis test was used to compare differences across quartile groups, and variables were described using medians (25th percentile, 75th percentile). For categorical variables, chi-square tests were employed to calculate *P*-values, and distributions were presented as counts (percentages). Soy isoflavone intake was adjusted for energy intake using the residual method. The resulting values represent relative intake levels independent of total energy. Residual-adjusted soy isoflavone intake was categorized into quartile groups (Q1, Q2, Q3, and Q4) using the following cutoff points: 8.68, 13.93, and 21.33 mg/d

*CKD* Chronic kidney disease, *BMI* Body mass index, *MET* Metabolic equivalent, *IQR* Interquartile range, *SBP* Systolic blood pressure, *DBP* Diastolic blood pressure, *FBG* Fasting blood glucose, *SUA* Serum uric acid

### The association between the prevalence of CKD and consumption of beans and bean products, as well as soy isoflavones

Participants who consumed the third, and fourth quartiles of total beans and bean products had a significantly lower prevalence of CKD after adjustments, as shown in Table 2. The ORs with 95% CIs were 0.92 (0.76–1.12), 0.71 (0.57–0.87), and 0.67 (0.54–0.82), respectively, with a linear trend (*P *_*for trend*_ < 0.001). Moreover, each SD increase in total beans and bean products residual-adjusted intake was significantly associated with a lower prevalence of CKD (OR = 0.86, 95% CI: 0.79–0.93). No statistically significant association was observed between plain bean residual-adjusted intake and CKD after adjustment for covariates. Higher residual-adjusted intakes of bean products were associated with a lower prevalence of CKD compared with the lowest quartile (Q2: OR = 0.79 [95% CI: 0.65–0.96]; Q3: OR = 0.72 [95% CI: 0.59–0.89]; Q4: OR = 0.63 [95% CI: 0.51–0.77]), with a significant linear trend (*P *_*for trend*_ < 0.001) and per SD increase in bean products was associated with 15% lower prevalence of CKD (OR = 0.85, 95% CI: 0.79–0.92). For soy isoflavone residual-adjusted intake, participants in the highest quartile had a 30% lower prevalence of CKD compared to the lowest quartile (OR = 0.70, 95% CI: 0.58–0.86), with a linear trend (*P *_*for trend*_ < 0.001) and each SD increase in soy isoflavone (OR = 0.86, 95% CI: 0.80–0.93). The similar results were also seen in daidzein, glycitein, and genistein consumption.

**Table 2:** Multivariate logistic regression analysis of residual-adjusted intake of beans and bean products, soy isoflavones and CKD

Variables	Residual-adjusted intake				*P* for trend	Continuous variable (every additional SD)^a^
	Q1	Q2	Q3	Q4		
Total beans and bean products (g/d)	< 44.77	44.77–68.47	68.48–100.91	≥ 100.92		
CKD/Without CKD	286/6464	244/6507	193/6558	177/6574		
Model 1	1.00 (reference)	0.85 (0.71, 1.01)	0.67 (0.55, 0.80)	0.61 (0.50, 0.74)	< 0.001	0.84 (0.77, 0.90)
Model 2	1.00 (reference)	0.85 (0.70, 1.03)	0.63 (0.51, 0.77)	0.59 (0.48, 0.71)	< 0.001	0.81 (0.75, 0.88)
Model 3	1.00 (reference)	0.92 (0.76, 1.12)	0.71 (0.57, 0.87)	0.67 (0.54, 0.82)	< 0.001	0.86 (0.79, 0.93)
Beans^b^ (g/d)	< 11.70	11.70–17.51	17.52–25.29	≥ 25.30		
CKD/Without CKD	226/6524	215/6536	220/6531	239/6512		
Model 1	1.00 (reference)	0.95 (0.79, 1.15)	0.97 (0.81, 1.18)	1.06 (0.88, 1.28)	0.411	1.02 (0.95, 1.08)
Model 2	1.00 (reference)	0.91 (0.74, 1.12)	0.89 (0.73, 1.09)	0.92 (0.76, 1.12)	0.510	0.98 (0.91, 1.05)
Model 3	1.00 (reference)	0.96 (0.78, 1.17)	0.93 (0.76, 1.15)	0.93 (0.76, 1.14)	0.520	0.97 (0.90, 1.05)
Bean products^c^ (g/d)	< 28.12	28.12–48.69	48.70–76.68	≥ 76.69		
CKD/Without CKD	306/6444	222/6529	199/6552	173/6578		
Model 1	1.00 (reference)	0.72 (0.60, 0.85)	0.64 (0.53, 0.77)	0.55 (0.46, 0.67)	< 0.001	0.81 (0.75, 0.88)
Model 2	1.00 (reference)	0.71 (0.58, 0.85)	0.62 (0.50, 0.75)	0.55 (0.45, 0.66)	< 0.001	0.80 (0.74, 0.87)
Model 3	1.00 (reference)	0.79 (0.65, 0.96)	0.72 (0.59, 0.89)	0.63 (0.51, 0.77)	< 0.001	0.85 (0.79, 0.92)
Soy isoflavones (mg/d)	< 8.68	8.68–13.92	13.93–21.32	≥ 21.33		
CKD/Without CKD	286/6464	222/6529	201/6550	191/6560		
Model 1	1.00 (reference)	0.77 (0.64, 0.92)	0.69 (0.58, 0.83)	0.66 (0.55, 0.79)	< 0.001	0.85 (0.79, 0.91)
Model 2	1.00 (reference)	0.76 (0.63, 0.92)	0.67 (0.54, 0.81)	0.63 (0.52, 0.77)	< 0.001	0.83 (0.77, 0.89)
Model 3	1.00 (reference)	0.83 (0.68, 1.01)	0.74 (0.60, 0.90)	0.70 (0.58, 0.86)	< 0.001	0.86 (0.80, 0.93)
Daidzein (mg/d)	< 1.41	1.41–2.31	2.32–3.44	≥ 3.45		
CKD/Without CKD	315/6435	245/6506	178/6573	162/6589		
Model 1	1.00 (reference)	0.77 (0.65, 0.91)	0.55 (0.46, 0.67)	0.50 (0.41, 0.61)	< 0.001	0.80 (0.74, 0.86)
Model 2	1.00 (reference)	0.73 (0.61, 0.88)	0.51 (0.41, 0.63)	0.47 (0.39, 0.58)	< 0.001	0.79 (0.72, 0.86)
Model 3	1.00 (reference)	0.77 (0.63, 0.94)	0.62 (0.49, 0.78)	0.58 (0.47, 0.72)	< 0.001	0.86 (0.79, 0.93)
Glycitein (mg/d)	< 0.77	0.77–1.60	1.61–2.67	≥ 2.68		
CKD/Without CKD	315/6435	237/6514	184/6567	164/6587		
Model 1	1.00 (reference)	0.74 (0.63, 0.88)	0.57 (0.48, 0.69)	0.51 (0.42, 0.62)	< 0.001	0.80 (0.74, 0.87)
Model 2	1.00 (reference)	0.68 (0.56, 0.83)	0.51 (0.41, 0.63)	0.48 (0.39, 0.59)	< 0.001	0.80 (0.73, 0.87)
Model 3	1.00 (reference)	0.77 (0.64, 0.91)	0.49 (0.39, 0.60)	0.45 (0.36, 0.57)	< 0.001	0.83 (0.76, 0.91)
Genistein (mg/d)	< 1.43	1.43–2.19	2.20–3.23	≥ 3.24		
CKD/Without CKD	279/6471	230/6521	209/6542	182/6569		
Model 1	1.00 (reference)	0.82 (0.68, 0.98)	0.74 (0.62, 0.89)	0.64 (0.53, 0.78)	< 0.001	0.85 (0.79, 0.91)
Model 2	1.00 (reference)	0.81 (0.67, 0.98)	0.72 (0.59, 0.88)	0.61 (0.50, 0.74)	< 0.001	0.83 (0.76, 0.89)
Model 3	1.00 (reference)	0.88 (0.72, 1.06)	0.79 (0.65, 0.97)	0.66 (0.55, 0.83)	< 0.001	0.86 (0.79, 0.93)

Model 1: crude (unadjusted) model;

Model 2: adjusted for age, sex, ethnicity, BMI, hypertension, diabetes, smoking status, drinking status, annual family income, education, physical activity, and energy intake;

Model 3: Model 2 additionally adjusted for dietary pattern (derived without beans and bean products)

*CKD* Chronic kidney disease, *OR* Odds ratio, *SD* Standard deviation, *CI* Confidence interval

^a^The SD values of total beans and bean products, beans, bean products, soy isoflavones, daidzein, glycitein, and genistein were 65.56, 14.70, 60.08, 13.68, 2.68, 2.80, and 1.91

^b^Beans include: soybeans, mung beans, various snap beans, bean sprouts, and other beans

^c^Bean products include: soy milk, soybean milk, tofu pudding, tofu, dried tofu sticks, and dried tofu

**Fig. 2:**
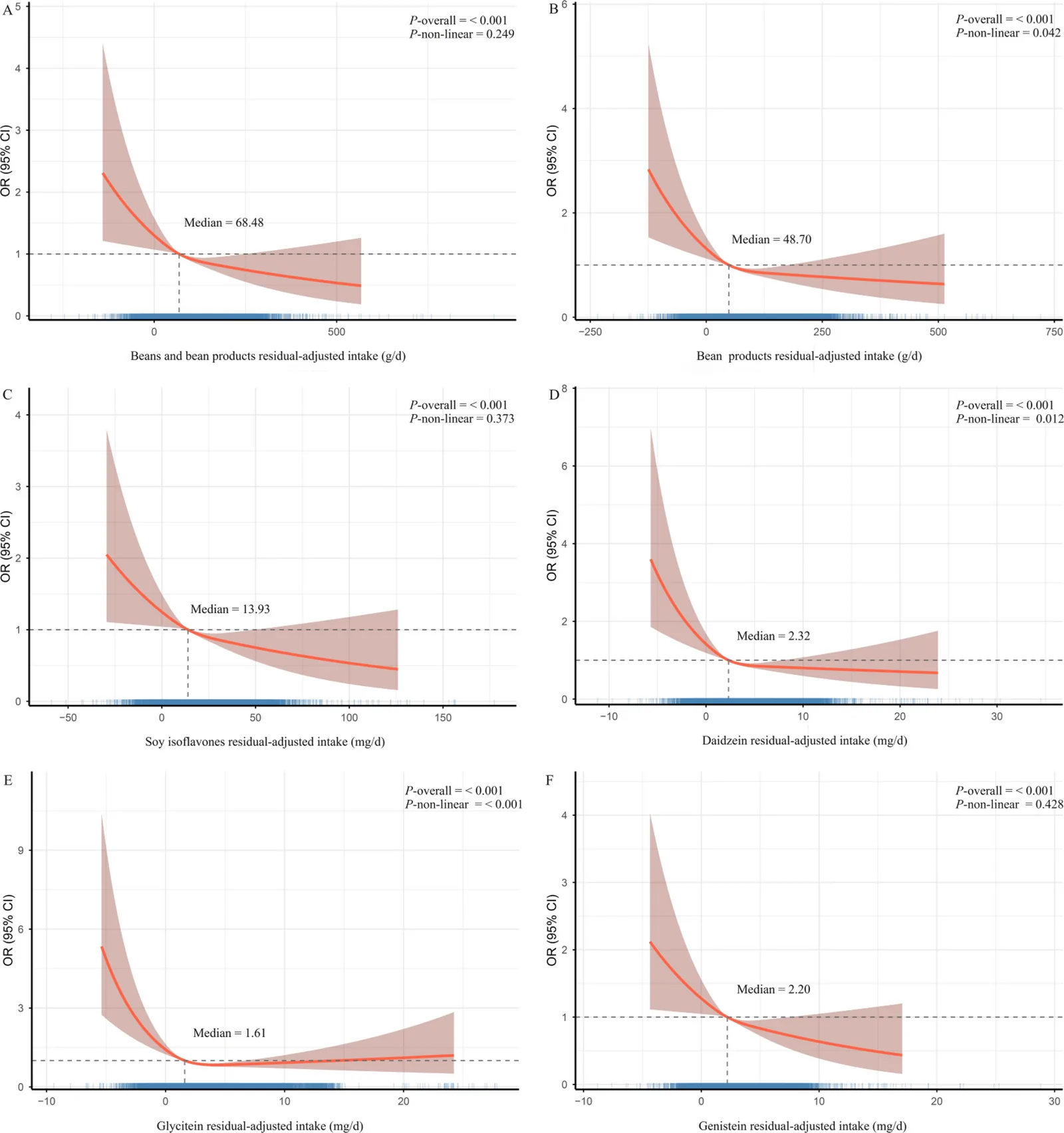
RCS of residual-adjusted intake of beans, bean products, and soy isoflavones with CKD. CKD, chronic kidney disease; CI, confidence interval; OR, odds ratio. ORs are indicated by orange lines, 95% CIs are indicated by light red area and blue area represents rug plot. RCS with three knots (10th, 50th, 90th percentiles) assessed nonlinear associations between residual-adjusted intake and CKD. The median of the residual-adjusted intake was used as the reference value in the RCS models. All models are adjusted according to age, sex, ethnicity, BMI, hypertension, diabetes, smoking status, drinking status, annual family income, education, physical activity, energy intake and dietary pattern (without beans and bean products)

RCS models were applied to characterize the non-linear relationships between the dietary factors and the prevalence of CKD, with the median residual-adjusted intake level set as the reference (Fig. 2). Significant overall associations were observed for all factors (all *P*-overall < 0.001). Notably, non-linear inverse associations were identified for the residual-adjusted intakes of bean products (*P*-non-linear = 0.042), daidzein (*P*-non-linear = 0.012), and glycitein (*P*-non-linear = 0.001). The fitted curves for these variables displayed a steep decrease in ORs at lower consumption levels, which then leveled off and plateaued at higher residual-adjusted intakes (L-shaped curve). In contrast, the associations of soy isoflavones (*P*-non-linear = 0.373), beans and bean products (*P*-non-linear = 0.249), and genistein (*P*-non-linear = 0.423) with the outcome demonstrated approximately linear trends. Due to the limited sample size, the uncertainty of the RCS model estimates significantly increased in the high residual-adjusted intake range. Therefore, the risk trend in this extremely high residual-adjusted intake interval should be interpreted with caution.

### Subgroup and sensitivity analyses

**Fig. 3:**
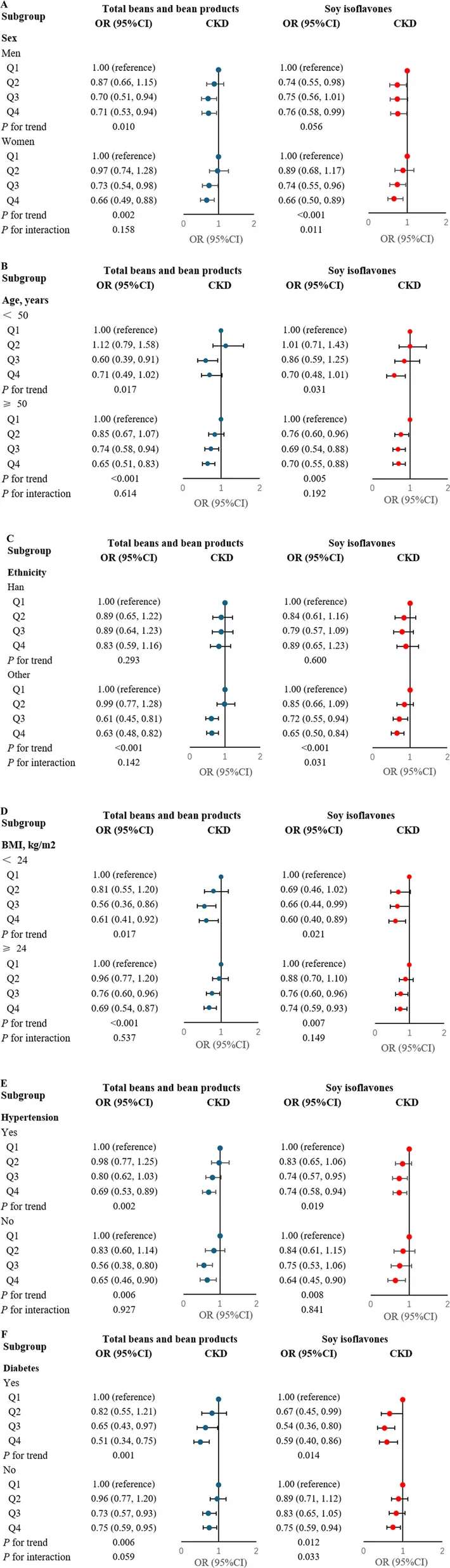
Subgroup analysis of associations between residual-adjusted intake of beans, bean products, soy isoflavones, and CKD. CKD, chronic kidney disease; OR, odds ratio; CI, confidence interval. The stratifications are (**A**) Sex, **B** Age, **C** Ethnicity, **D** BMI, **E** Hypertension, and **F** Diabetes. The blue dots and horizontal bars represent the OR and 95% CI for total beans and bean products, while the red dots and bars represent those for soy isoflavones. Q1 was treated as the reference group. ORs and 95% CIs were calculated using a multivariable logistic regression analysis model with adjustment for age, sex, ethnicity, BMI, hypertension, diabetes, smoking status, drinking status, annual family income, education, physical activity, energy intake and dietary pattern (without beans and bean products). Stratification variables were omitted from the adjustment set in the corresponding stratified analyses

Figure 3 illustrates the subgroup analyses. For total beans and bean products, significant linear inverse trends with CKD prevalence were observed in men (*P *_*for trend*_ = 0.010), women (*P *_*for trend*_ = 0.002), participants aged ≥ 50 years (*P *_*for trend*_ = < 0.001), other ethnicity (*P *_*for trend*_ = < 0.001), BMI ≥ 24 kg/m^2^ (*P *_*for trend*_ = < 0.001), and those with or without hypertension (*P *_*for trend*_ = 0.002 and 0.006, respectively) or diabetes (*P *_*for trend*_ = 0.001 and 0.006, respectively). However, the trends did not reach the Bonferroni-adjusted significance level in participants aged < 50 years (*P *_*for trend*_ = 0.017), Han ethnicity (*P *_*for trend*_ = 0.293), or those with BMI < 24 kg/m^2^ (*P *_*for trend*_ = 0.017). Furthermore, no significant interactions were observed across any predefined strata (all *P* for interaction > 0.0167), suggesting a consistent protective effect across populations.

In contrast, soy isoflavones exhibited distinct heterogeneity across subgroups. Significant inverse associations were found in women (*P *_*for trend*_ = < 0.001), participants aged ≥ 50 years (*P *_*for trend*_ = 0.005), BMI ≥ 24 kg/m^2^ (*P *_*for trend*_ = 0.007), other ethnicity (*P *_*for trend*_ = < 0.001), those without hypertension (*P *_*for trend*_ = 0.008), and those with or without diabetes (*P *_*for trend*_ = 0.014 and 0.012, respectively). Conversely, no significant trends were observed in men, participants aged < 50 years, Han ethnicity, BMI < 24 kg/m^2^, or those with hypertension (all *P *_*for trend*_ = > 0.0167). Importantly, sex was identified as a significant effect modifier for the association between soy isoflavones and CKD (*P* for interactio*n* = 0.011), indicating a pronounced nephroprotective benefit in females. No other significant effect modifications were found across the remaining strata (all *P* for interaction > 0.0167). The Supplementary Table 3 displays the number of participants and CKD cases in each subgroup. The table shows that even the smallest stratum still contains an adequate number of events to support stable estimation, and the interaction *P* values are therefore not driven by small-sample artifacts. No significant interactions were found in the remaining subgroup analyses (Supplementary Fig. 1 and Fig. 2).

In sensitivity analyses, the results remained consistent when: (1) Excluding only participants under 40 years (rather than all under 18); (2) Removing individuals with recent dietary changes (during the previous year); (3) Additionally adjusting for hyperuricemia in multivariate regression models; (4) Using the K/DOQI guidelines established by the National Kidney Foundation as diagnostic criteria for CKD.‌ (5) Protein, sodium, and red meat intake were adjusted for by including them in multivariate regression models. (6) Using urine protein positivity as the outcome, excluding participants with isolated reduced eGFR. (7) Using reduced eGFR as the outcome, excluding participants with isolated urine protein positivity (Supplementary Table 4).

## Discussion

### The relationship between beans and bean products, soy isoflavones, and CKD

After applying the inclusion and exclusion criteria, 27,003 participants were included in this study. Inverse nonlinear and dose–response associations were observed between total beans and bean products intake, soy isoflavone consumption, and CKD prevalence. Although plain beans intake was not associated with CKD, higher intake of beans and bean products showed a significant inverse association, consistent with findings from a cross-sectional study of 20,733 rural adults in Henan province [13], although its rural only population limits generalizability. Although previous studies share similarities with this study in terms of included independent variables, CKD definitions, and focus on CKD prevalence, they did not conduct analyses stratified by processing status and not using nutrient residual method to correct intake and exclude the influence of energy. Importantly, the present study extends these findings by also investigating the association between soy isoflavones and CKD. Notably, this is the first study to examine the association between soy isoflavone intake and CKD in a Chinese population. Additionally, multiplicative interactions were observed between soy isoflavones and sex in relation to CKD.

### Potential biological mechanisms of beans intake on CKD prevalence

Extensive research has demonstrated that bean-based foods exert protective effects against several chronic diseases, including hypertension, diabetes, hyperuricemia, and certain cancers [37–39]. These beneficial effects are primarily attributed to their nutritional composition and bioactive compounds, as bean-based foods are rich in high-quality plant proteins that supply all essential amino acids [40]. Compared with animal proteins, beans contain lower levels of cholesterol and fewer potentially harmful components [41]. Accumulating epidemiological evidence has demonstrated that increased intake of plant protein significantly reduces the risk of CKD, although an increased intake of animal protein was not observed to elevate CKD risk. Notably, replacing 3% of energy intake from animal protein with plant protein was associated with an 80% lower odds of CKD (OR = 0.20, 95% CI: 0.06–0.63) [42]. This effect may be due to animal proteins producing greater amounts of amino and sulfhydryl groups—pro-inflammatory substances—than plant proteins, contributing to renal damage through inflammatory responses, oxidative stress, gut microbiota dysregulation, increased metabolic burden, and impaired renal function [43]. Moreover, bean-based foods are rich in unsaturated fatty acids, dietary fiber, and micronutrients such as iron and calcium [44], and previous research has shown that a higher intake of dietary fiber is significantly associated with a lower prevalence of CKD relative to a lower intake (OR = 0.68, 95% CI: 0.49–0.94). This benefit is largely driven by the ability of dietary fiber to enhance gut motility, attenuate postprandial glycemic excursions, and reduce the production of pro-inflammatory uremic toxins from the gut, thereby conferring renoprotective advantages [45].

To date, no study has specifically examined the association between the intake of plain beans or bean products alone and CKD. In this study, the intake of plain beans was not significantly associated with CKD, whereas consumption of bean products—including soy milk, soybean milk, tofu pudding, tofu, dried tofu sticks and dried tofu—was associated with a considerable reduction in CKD prevalence. This may primarily be explained by processing methods, such as those used in tofu production, which remove most antinutrient components while improving nutrient absorption and increasing protein content through soaking and refinement procedures [46]. Furthermore, bean milk contains high levels of folate and vitamin B12, and processing techniques such as heating and fermentation reduce harmful substances (e.g., biogenic amines and allergens) while decreasing the bioavailability of antinutrients [47, 48]. Therefore, substituting red meat with bean-based protein—particularly by increasing the intake of bean products—may represent a promising dietary strategy for lowering CKD prevalence.

### Potential biological mechanisms of soy isoflavone intake on CKD pathogenesis

Soy isoflavones, the main bioactive compounds in beans and bean products, exert notable renal protective effects that deserve attention. However, the independent association between soy isoflavone intake and CKD has not been previously investigated. Soy isoflavones predominantly consist of three types: glycitein, daidzein, and genistein [49], which share structural similarities with estrogen. In beans and their products, soy isoflavones are mainly present as β-glycosides and their corresponding acetyl and malonyl esters [50]. In vitro studies indicate that isoflavones exert antioxidant and anti-inflammatory effects by modulating the expression of tumor necrosis factor-α (TNF-α), interleukins (IL-6, IL-8, IL-1β), prostaglandin E2 (PGE2), monocyte chemoattractant protein-1, and intercellular adhesion molecule-1 [51, 52]. Moreover, animal studies reveal that peroxisome proliferator-activated receptor γ (PPAR-γ) in the kidney directly regulates metabolic genes, thereby influencing lipid and glucose metabolism [53], and isoflavones mediate renal benefits by modulating PPAR-γ expression [54]. Cellular evidence further indicates that disruption of the renin-angiotensin system (RAS)—a key regulator of blood pressure, fluid volume, and electrolyte balance—through increased renal angiotensinogen (AGT) secretion can contribute to the development of renal disease. Notably, isoflavones protect renal structure and function by inhibiting hydrogen peroxide induced AGT secretion in proximal tubular epithelial cells [55]. Genistein, one of the most important types of soy isoflavones, has been found to reduce renal inflammation and fibrosis by blocking Toll-like receptor-4 (TLR-4)-mediated activation of the nuclear factor-kappaB (NF-κB) and extracellular signal-regulated kinase (ERK) pathways, thereby inhibiting the transcription of inflammatory and fibrotic genes [56, 57]. However, these findings are largely based on mechanistic studies, and large-scale observational research is needed to validate the association between soy isoflavone intake and CKD.

### Analysis of differences between subgroups

Subgroup analyses indicated that higher intake of beans, bean products, and soy isoflavones was inversely associated with CKD across all subgroups, except when stratified by ethnicity. Participants in this study were recruited from Northeast China, including Liaoning, Jilin, Heilongjiang, and Inner Mongolia provinces, with Han and non-Han ethnic groups mainly consisting of Manchu, Korean, Mongolian, and other minorities. The primary reason may be that the prevalence of CKD was higher in the non-Han population (4.15%) than in the Han population (2.63%), and the dietary intakes of beans, bean products, and soy isoflavones were higher in the non-Han subgroup (*P* < 0.001). Additionally, non-Han individuals are more likely to reside in rural areas, where the natural environment is considerably better than that of Han-populated regions (characterized by lower PM_2.5_ concentrations). Previous studies have reported that exposure to higher PM_2.5_ concentrations is associated with elevated albuminuria levels and an increased risk of CKD incidence in cohorts [58]. This might suggest that environmental factors could attenuate the inverse association between bean residual-adjusted intake and CKD prevalence among the Han population. Furthermore, the Han population generally exhibits stronger health awareness and possesses more comprehensive knowledge regarding CKD prevention. In addition to bean consumption, they might actively adopt other preventive measures, which could potentially obscure the association between bean residual-adjusted intake and CKD in this subgroup. Conversely, ethnic minorities tend to have relatively weaker health awareness, thereby making the lower prevalence between bean adjusted intake intake and CKD more readily discernible. Collectively, these factors may explain why the prevalence reduction associated with increased adjusted intake intake was more pronounced in non-Han populations than in the Han population. However, due to the paucity of relevant research, there is currently no definitive explanation for these ethnic disparities, and further well-designed studies are warranted to elucidate the underlying reasons for this observed difference.

Notably, we observed a significant multiplicative interaction between sex and residual-adjusted soy isoflavone intake with respect to CKD prevalence (*P* for interactio*n* = 0.011). Stratified analyses revealed a robust inverse association in women, but not in men, indicating that the nephroprotective effect of soy isoflavones is sex-dependent. This disparity can be biologically attributed to the phytoestrogenic properties of isoflavones. Structurally similar to estradiol, isoflavones may exert anti-inflammatory and vascular protective effects via estrogen receptor activation, which may be attenuated by the testosterone milieu in males [59]. Furthermore, sex dimorphism in mitochondrial function may play a crucial role. Males exhibit lower mitochondrial respiratory capacity and inferior antioxidant defense systems, manifesting as increased mitochondrial fragmentation [60]. Given that disruption of mitochondrial homeostasis is central to the pathogenesis of kidney injury, these subcellular differences could explain the diminished benefits observed in males, a phenomenon consistently corroborated by animal models of AKI [61].

### Advantages and disadvantages of this study

This study is the first large-scale cross-sectional investigation to examine the association between soy isoflavone intake and CKD, while independently assessing the associations of plain beans and bean products with CKD. The strengths of this study include: (1) A large sample size with comprehensive baseline data allowing for adjustment for multiple confounders; (2) Restricted cubic spline functions were employed to provide a more detailed characterization of the associations between the groups and CKD, demonstrating the nonlinear relationships among them; (3) Seven sensitivity analyses that confirmed the robustness of the findings. However, several limitations should be acknowledged: (1) As a cross-sectional study design, causal relationships cannot be inferred, requiring validation in prospective cohorts; (2) Residual unmeasured confounding may remain, and grouping beans and bean products restricts the identification of the most protective food types; (3) The study population was restricted to Northeast China, which may limit generalizability to other regions and ethnic groups. (4) This study is subject to selection bias due to missing data. Specifically, data on proteinuria and serum uric acid were missing for 4,488 (12.34%) and 2,624 (7.21%) participants, respectively. Baseline comparisons showed differences between excluded and included participants across several characteristics: the excluded group had higher proportions of ethnic minorities, individuals with low physical activity, and obese individuals; lower total energy and multiple nutrient intakes; but higher intakes of beans and their products, as well as soy isoflavones and their subtypes. This suggests that the missing data were not completely random and may have introduced selection bias. Such differences may cause the results to deviate from the true effect. Moreover, these characteristic differences also limit the generalizability of the conclusions to ethnic minority populations, those with low physical activity, and obese individuals. Notwithstanding these limitations, the observed negative nonlinear associations between beans and bean products intake, soy isoflavones, and CKD prevalence among this population offer novel insights for CKD prevention strategies (Supplementary Table 5). (5) Although dietary pattern scores derived from EFA represent a data-driven approach that can control for confounding from dietary factors, this method is unable to model specific macronutrient substitutions. (6) According to current guidelines, the diagnosis of CKD requires that reduced kidney function persist for at least three months. The reliance of this study on a single assessment may lead to non-differential misclassification, particularly because it is difficult to distinguish transient proteinuria or acute kidney injury caused by short-term creatinine fluctuations from sustained CKD. Moreover the inclusion of random urine samples may have introduced variability, as random samples are more susceptible to false-positive results from orthostatic or transient proteinuria. As a result, some individuals with temporary renal dysfunction may be erroneously classified as having CKD.

## Conclusion

In summary, intake of beans and bean products, as well as soy isoflavones, showed an inverse nonlinear association with CKD prevalence. A multiplicative interaction effect was observed between sex and residual-adjusted soy isoflavone intake in relation to CKD. These findings suggest an inverse association between the consumption of beans, bean products—particularly processed bean products—and soy isoflavones and CKD prevalence, providing useful clues for future dietary guidelines. An appropriate increase in the intake of these foods might be considered as part of a healthy dietary pattern. Nevertheless, these associations require confirmation in future prospective studies.

## Supplementary Information

Supplementary Material 1.

## Data Availability

Data described in the manuscript, code book, and analytic code will be made available upon request pending application and approval.

## Abbreviations

AGT: Angiotensinogen
AKI: Acute Kidney Injury
BMI: Body mass index
CI: Confidence intervals
CKD: Chronic kidney disease
DALYs: Disability-adjusted life years
EFA: Exploratory factor analysis
eGFR: Estimated glomerular filtration rate
ERK: Extracellular signal-regulated kinase
ESRD: End-stage renal disease
FFQ: Food frequency questionnaire
IL: Interleukins
MET-h/wk: Metabolic equivalent hours per week
NF-κB: Nuclear factor-kappaB
NEC-biobank: Northeast of China biobank
OR: Odds ratios
PPAR-γ: Peroxisome proliferator-activated receptor γ
PGE2: Prostaglandin E2
RAS: Renin-angiotensin system
RCS: Restricted cubic spline
RRT: Renal replacement therapy
TLR-4: Toll-like receptor-4
TNF-α: Tumor necrosis factor-α
VIF: Variance inflation factors

## References

[1] Lv JC, Zhang LX. Prevalence and Disease Burden of Chronic Kidney Disease. Adv Exp Med Biol. 2019;1165:3–15. 10.1007/978-981-13-8871-2_1 31399958

[2] Alkhatib L, Velez Diaz LA, Varma S, Chowdhary A, Bapat P, Pan H, et al. Lifestyle Modifications and Nutritional and Therapeutic Interventions in Delaying the Progression of Chronic Kidney Disease: A Review. Cureus. 2023;15(2):e34572. 10.7759/cureus.34572 PMC9981552 36874334

[3] Luyckx VA, Tuttle KR, Garcia-Garcia G, Gharbi MB, Heerspink HJL, Johnson DW, et al. Reducing major risk factors for chronic kidney disease. Kidney Int Suppl (2011). 2011;7(2):71–87. 10.1016/j.kisu.2017.07.003 PMC6341126 30675422

[4] Dong B, Zhao Y, Wang J, Lu C, Chen Z, Ma R, et al. Epidemiological analysis of chronic kidney disease from 1990 to 2019 and predictions to 2030 by Bayesian age-period-cohort analysis. Ren Fail. 2024;46(2):2403645. 10.1080/0886022X.2024.2403645 PMC11413963 39297199

[5] Zhu W, Han M, Wang Y, Wang G. Trend analysis and prediction of the incidence and mortality of CKD in China and the US. BMC Nephrol. 2024;25(1):76. 10.1186/s12882-024-03518-w PMC10908046 38429632

[6] Mok Y, Ballew SH, Matsushita K. Chronic kidney disease measures for cardiovascular risk prediction. Atherosclerosis. 2021;335:110–8. 10.1016/j.atherosclerosis.2021.09.007 34556333

[7] Spence JD, Urquhart BL. Cerebrovascular Disease, Cardiovascular Disease, and Chronic Kidney Disease: Interplays and Influences. Curr Neurol Neurosci Rep. 2022;22(11):757–66. 10.1007/s11910-022-01230-6 36181576

[8] Wang L, Xu X, Zhang M, Hu C, Zhang X, Li C, et al. Prevalence of Chronic Kidney Disease in China: Results From the Sixth China Chronic Disease and Risk Factor Surveillance. JAMA Intern Med. 2023;183(4):298–310. 10.1001/jamainternmed.2022.6817 PMC9941971 36804760

[9] Yang C, Wang H, Zhao X, Matsushita K, Coresh J, Zhang L, et al. CKD in China: Evolving Spectrum and Public Health Implications. Am J Kidney Dis. 2020;76(2):258–64. 10.1053/j.ajkd.2019.05.032 31492486

[10] Chen LR, Chen KH. Utilization of isoflavones in soybeans for women with menopausal syndrome: an overview. Int J Mol Sci. 2021;22(6):3212. 10.3390/ijms22063212 PMC8004126 33809928

[11] Fan Y, Wang M, Li Z, Jiang H, Shi J, Shi X, et al. Intake of Soy, Soy Isoflavones and Soy Protein and Risk of Cancer Incidence and Mortality. Front Nutr. 2022;9:847421. 10.3389/fnut.2022.847421 PMC8931954 35308286

[12] Barańska A, Błaszczuk A, Polz-Dacewicz M, Kanadys W, Malm M, Janiszewska M, et al. Effects of Soy Isoflavones on Glycemic Control and Lipid Profile in Patients with Type 2 Diabetes: A Systematic Review and Meta-Analysis of Randomized Controlled Trials. Nutrients. 2021;13(6):1886. 10.3390/nu13061886 PMC8229139 34072748

[13] Yin L, Dong X, Liao W, Liu X, Zheng Z, Liu D, et al. Relationships of beans intake with chronic kidney disease in rural adults: A large-scale cross-sectional study. Front Nutr. 2023;10:1117517. 10.3389/fnut.2023.1117517 PMC10111024 37081921

[14] Liu ZM, Ho SC, Chen YM, Tang N, Woo J. Effect of whole soy and purified isoflavone daidzein on renal function–a 6-month randomized controlled trial in equol-producing postmenopausal women with prehypertension. Clin Biochem. 2014;47(13–14):1250–6. 10.1016/j.clinbiochem.2014.05.054 24877660

[15] Gholami A, Darudi F, Baradaran HR, Hariri M. Effect of soy isoflavones on C-reactive protein in chronic inflammatory disorders. Int J Vitam Nutr Res. 2023;93(5):447–58. 10.1024/0300-9831/a000751 35291882

[16] Mitsunami M, Mínguez-Alarcón L, Florio AA, Wang S, Attaman JA, Souter I, et al. Intake of soy products and soy isoflavones in relation to ovarian reserve. Fertil Steril. 2023;119(6):1017–29. 10.1016/j.fertnstert.2023.02.039 PMC10225319 36871859

[17] Boutas I, Kontogeorgi A, Dimitrakakis C, Kalantaridou SN. Soy isoflavones and breast cancer risk: a meta-analysis. In Vivo. 2022;36(2):556–62. 10.21873/invivo.12737 PMC8931889 35241506

[18] Zhixiang Y, Yongxing X, Juan L, Qing Y, Yangyang L, Zhangrui Z, et al. The effects of soy protein and soy isoflavones intake on chronic kidney disease: a systematic review and meta-analysis. Int Urol Nephrol. 2025;57(5):1533–53. 10.1007/s11255-024-04301-4 39611889

[19] Ahmed MS, Calabria AC, Kirsztajn GM. Short-term effects of soy protein diet in patients with proteinuric glomerulopathies. J Bras Nefrol. 2011;33(2):150–9. 10.1590/s0101-28002011000200006 21789429

[20] Chang Q, Wu Q, Xia Y, Zhang H, Gao S, Zhang Y, et al. Cohort Profile: The Northeast China Biobank (NEC-Biobank). Int J Epidemiol. 2023;52(2):e125–36. 10.1093/ije/dyac172 36018264

[21] Hu FB, Stampfer MJ, Rimm E, Ascherio A, Rosner BA, Spiegelman D, et al. Dietary fat and coronary heart disease: a comparison of approaches for adjusting for total energy intake and modeling repeated dietary measurements. Am J Epidemiol. 1999;149(6):531–40. 10.1093/oxfordjournals.aje.a009849 10084242

[22] Shi Z, Taylor AW, Riley M, Byles J, Liu J, Noakes M. Association between dietary patterns, cadmium intake and chronic kidney disease among adults. Clin Nutr. 2018;37(1):276–84. 10.1016/j.clnu.2016.12.025 28094058

[23] Cui Q, Xia Y, Liu Y, Sun Y, Ye K, Li W, et al. Validity and reproducibility of a FFQ for assessing dietary intake among residents of northeast China: northeast cohort study of China. Br J Nutr. 2023;129(7):1252–65. 10.1017/S0007114522002318 35912695

[24] Yang Y, Wang G, Pan X. Chinese food composition table. 6th ed. Beijing: Peking University Medical Press; 2019.

[25] Hu EA, Rebholz CM. Can Dietary Patterns Modify Risk for CKD? Clin J Am Soc Nephrol. 2019;14(10):1419–20. 10.2215/CJN.09440819 PMC6777597 31591254

[26] Chen S, Hu S, Shen S, Zhang J, Xu X, Yu M, et al. Dietary patterns and cardiovascular diseases among Chinese high-risk population aged 35 years and older: A 6-year cohort study. Curr Res Food Sci. 2025;10:100960. 10.1016/j.crfs.2024.100960 PMC11729030 39807360

[27] Pei Z, Zhang J, Qin W, Hu F, Zhao Y, Zhang X, et al. Association between Dietary Patterns and Depression in Chinese Older Adults: A Longitudinal Study Based on CLHLS. Nutrients. 2022;14(24):5230. 10.3390/nu14245230 PMC9782901 36558386

[28] Hashimoto Y, Imaizumi T, Kato S, Yasuda Y, Ishimoto T, Kawashiri H, et al. Effect of body mass index on the association between alcohol consumption and the development of chronic kidney disease. Sci Rep. 2021;11(1):20440. 10.1038/s41598-021-99222-y PMC8516880 34650098

[29] Miller WG, Kaufman HW, Levey AS, Straseski JA, Wilhelms KW, Yu HE, et al. National Kidney Foundation Laboratory Engagement Working Group Recommendations for Implementing the CKD-EPI 2021 Race-Free Equations for Estimated Glomerular Filtration Rate: Practical Guidance for Clinical Laboratories. Clin Chem. 2022;68(4):511–20. 10.1093/clinchem/hvab278 34918062

[30] Bennett DA, Du H, Clarke R, Guo Y, Yang L, Bian Z, et al. Association of physical activity with risk of major cardiovascular diseases in Chinese men and women. JAMA Cardiol. 2017;2(12):1349–58. 10.1001/jamacardio.2017.4069 PMC5814992 29117341

[31] Chobanian AV, Bakris GL, Black HR, Cushman WC, Green LA, Izzo JL Jr, et al. The Seventh Report of the Joint National Committee on Prevention, Detection, Evaluation, and Treatment of High Blood Pressure: the JNC 7 report. JAMA. 2003;289(19):2560–72. 10.1001/jama.289.19.2560 12748199

[32] Liu C, Yang C, Zhao Y, Ma Z, Bi J, Liu Y, et al. Associations between long-term exposure to ambient particulate air pollution and type 2 diabetes prevalence, blood glucose and glycosylated hemoglobin levels in China. Environ Int. 2016;92–93:416–21. 10.1016/j.envint.2016.03.028 PMC4902714 27148900

[33] Sun M, Lyu Z, Wang C, Li Y, Zhao D, Ran X, et al. 2024 Update of Chinese Guidelines for Diagnosis and Treatment of Hyperuricemia and Gout Part I: Recommendations for General Patients. Int J Rheum Dis. 2025;28(7):e70375. 10.1111/1756-185x.70375 PMC12280528 40692263

[34] Wang QQ, Yu SC, Qi X, Hu YH, Zheng WJ, Shi JX, et al. Overview of logistic regression model analysis and application. Zhonghua Yu Fang Yi Xue Za Zhi. 2019;53(9):955–60. 10.3760/cma.j.issn.0253-9624.2019.09.018 31474082

[35] Desquilbet L, Mariotti F. Dose-response analyses using restricted cubic spline functions in public health research. Stat Med. 2010;29(9):1037–57. 10.1002/sim.3841 20087875

[36] Park JH, Jo YI, Lee JH. Renal effects of uric acid: hyperuricemia and hypouricemia. Korean J Intern Med. 2020;35(6):1291–304. 10.3904/kjim.2020.410 PMC7652664 32872730

[37] Mosallanezhad Z, Mahmoodi M, Ranjbar S, Hosseini R, Clark CCT, Carson-Chahhoud K, et al. Soy intake is associated with lowering blood pressure in adults: a systematic review and meta-analysis of randomized double-blind placebo-controlled trials. Complement Ther Med. 2021;59:102692. 10.1016/j.ctim.2021.102692 33636295

[38] Piao W, Li S, Guo Q, Cheng X, Xu X, Zhao L, et al. Bean and Nut Intake Were Protective Factors for Comorbid Hypertension and Hyperuricemia in Chinese Adults: Results from China Nutrition and Health Surveillance (2015–2017). Nutrients. 2024;16(2):192. 10.3390/nu16020192 PMC10820914 38257085

[39] Zuo X, Zhao R, Wu M, Wan Q, Li T. Soy Consumption and the Risk of Type 2 Diabetes and Cardiovascular Diseases: A Systematic Review and Meta-Analysis. Nutrients. 2023;15(6):1358. 10.3390/nu15061358 PMC10058927 36986086

[40] Mcgraw NJ, Krul ES, Grunz-Borgmann E, Parrish AR. Soy-based renoprotection. World J Nephrol. 2016;5(3):233–57. 10.5527/wjn.v5.i3.233 PMC4848148 27152261

[41] Naghshi S, Sadeghi O, Willett WC, Esmaillzadeh A. Dietary intake of total, animal, and plant proteins and risk of all cause, cardiovascular, and cancer mortality: systematic review and dose-response meta-analysis of prospective cohort studies. BMJ. 2020;370:m2412. 10.1136/bmj.m2412 PMC7374797 32699048

[42] Joshi S, Hashmi S, Shah S, Kalantar-Zadeh K. Plant-based diets for prevention and management of chronic kidney disease. Curr Opin Nephrol Hypertens. 2020;29(1):16–21. 10.1097/MNH.0000000000000574 31725014

[43] Ko GJ, Rhee CM, Kalantar-Zadeh K, Joshi S. The Effects of High-Protein Diets on Kidney Health and Longevity. J Am Soc Nephrol. 2020;31(8):1667–79. 10.1681/ASN.2020010028 PMC7460905 32669325

[44] Rizzo G, Baroni L. Soy, soy foods and their role in vegetarian diets. Nutrients. 2018;10(1):43. 10.3390/nu10010043 PMC5793271 29304010

[45] Kim H, Rebholz CM. Plant-based diets for kidney disease prevention and treatment. Curr Opin Nephrol Hypertens. 2024;33(6):593–602. 10.1097/MNH.0000000000001015 PMC11419939 39115418

[46] Guan X, Zhong X, Lu Y, Du X, Jia R, Li H, et al. Changes of Soybean Protein during Tofu Processing. Foods. 2021;10(7):1594. 10.3390/foods10071594 PMC8306988 34359464

[47] Collard KM, Mccormick DP. A nutritional comparison of cow’s milk and alternative milk products. Acad Pediatr. 2021;21(6):1067–9. 10.1016/j.acap.2020.12.007 33373745

[48] Mollakhalili-Meybodi N, Arab M, Zare L. Harmful compounds of soy milk: characterization and reduction strategies. J Food Sci Technol. 2022;59(10):3723–32. 10.1007/s13197-021-05249-4 PMC9525506 36193379

[49] Akaza H. Prostate cancer chemoprevention by soy isoflavones: role of intestinal bacteria as the “second human genome.” Cancer Sci. 2012;103(6):969–75. 10.1111/j.1349-7006.2012.02257.x PMC7685082 22372745

[50] Setchell KD, Cole SJ. Variations in isoflavone levels in soy foods and soy protein isolates and issues related to isoflavone databases and food labeling. J Agric Food Chem. 2003;51(14):4146–55. 10.1021/jf026199b 12822960

[51] Medjakovic S, Mueller M, Jungbauer A. Potential health-modulating effects of isoflavones and metabolites via activation of PPAR and AhR. Nutrients. 2010;2(3):241–79. 10.3390/nu2030241 PMC3257647 22254019

[52] Blay M, Espinel AE, Delgado MA, Baiges I, Bladé C, Arola L, et al. Isoflavone effect on gene expression profile and biomarkers of inflammation. J Pharm Biomed Anal. 2010;51(2):382–90. 10.1016/j.jpba.2009.03.028 19410411

[53] Corrales P, Izquierdo-Lahuerta A, Medina-Gómez G. Maintenance of Kidney Metabolic Homeostasis by PPAR Gamma. Int J Mol Sci. 2018;19(7):2063. 10.3390/ijms19072063 PMC6073436 30012954

[54] Pessoa EA, Convento MB, Castino B, Leme AM, De Oliveira AS, Aragão A, et al. Beneficial Effects of Isoflavones in the Kidney of Obese Rats Are Mediated by PPAR-Gamma Expression. Nutrients. 2020;12(6):1624. 10.3390/nu12061624 PMC7352183 32492810

[55] Kamiyama M, Adachi H, Ogiwara M, Ishikawa M, Inoue S, Iwata M, et al. Isoflavones inhibit hydrogen peroxide-induced angiotensinogen secretion. Int J Mol Sci. 2025;26(9):4029. 10.3390/ijms26094029 PMC12071920 40362266

[56] Vallés PG, Gil Lorenzo AF, Garcia RD, Cacciamani V, Benardon ME, Costantino VV. Toll-like Receptor 4 in Acute Kidney Injury. Int J Mol Sci. 2023;24(2):1415. 10.3390/ijms24021415 PMC9864062 36674930

[57] Peng Q, Li Y, Shang J, Huang H, Zhang Y, Ding Y, et al. Effects of Genistein on Common Kidney Diseases. Nutrients. 2022;14(18):3768. 10.3390/nu14183768 PMC9506319 36145144

[58] Al-Aly Z, Bowe B. Air Pollution and Kidney Disease. Clin J Am Soc Nephrol. 2020;15(3):301–3. 10.2215/CJN.16031219 PMC7057298 32125277

[59] O’brien Z, Cass A, Cole L, Finfer S, Gallagher M, Mcarthur C, et al. Sex and mortality in septic severe acute kidney injury. J Crit Care. 2019;49:70–6. 10.1016/j.jcrc.2018.10.017 30388491

[60] Cardinale DA, Larsen FJ, Schiffer TA, Morales-Alamo D, Ekblom B, Calbet JaL, et al. Superior intrinsic mitochondrial respiration in women than in men. Front Physiol. 2018;9:1133. 10.3389/fphys.2018.01133 PMC6108574 30174617

[61] Ma HY, Chen S, Du Y. Estrogen and estrogen receptors in kidney diseases. Ren Fail. 2021;43(1):619–42. 10.1080/0886022X.2021.1901739 PMC8018493 33784950

