# Supplementary material for: Associations of beans, bean products, and soy isoflavone intake with chronic kidney disease: a cross-sectional study
Source: Nutr J. 2026 Aug 5;25:119. doi: 10.1186/s12937-026-01380-0 (PMC13726222; doi:10.1186/s12937-026-01380-0)
Supplement: Supplementary file 1 [file 12937_2026_1380_MOESM1_ESM.pdf]

# Associations of Beans, Bean Products, and Soy Isoflavone Intake with Chronic Kidney Disease: A Cross-Sectional Study

Song Zhang [1,2†], Chao Ji [1†], Yang Xia [1*], Yuhong Zhao [1,3*]

**Authors' Affiliations:**

1. Department of Clinical Epidemiology, Shengjing Hospital of China Medical University, Shenyang, China.

2. School of Public Health, China Medical University, Shenyang, China.

3. Liaoning Key Laboratory of Precision Medical Research on Major Chronic Disease, Shenyang, China.

† Song Zhang and Chao Ji contributed equally to this work

* Corresponding Authors: Yang Xia, and Yuhong Zhao.

**Supplementary Table 1** Specific variables and proportion of missing data.

**Supplementary Table 2** Baseline characteristics of study participants stratified by Beans and bean products residual-adjusted intake.

**Supplementary Table 3** Subgroup analysis of the association between Soy isoflavones residual-adjusted intake and CKD.

**Supplementary Figure 1** Subgroup analysis of associations of residual-adjusted intake of bean products, daidzein, glycitein, genistein with CKD. CKD, chronic kidney disease; OR, odds ratio; CI, confidence interval. The stratifications are (A) sex, (B) age, (C) ethnicity, (D) BMI, (E) hypertension, and (F) diabetes. The blue, red, yellow, and green dots and horizontal bars represent the ORs and 95% CIs for bean products, daidzein, glycitein, and genistein, respectively. The Q1 group served as the reference group. ORs and 95% CIs were calculated using a multivariable multivariate logistic regression analysis model with adjustment for age, sex, ethnicity, BMI, hypertension, diabetes, smoking status, drinking status, annual family income, education, physical activity, energy intake and dietary pattern (without beans and bean products). Stratification variables were omitted from the adjustment set in the corresponding stratified analyses.

**Supplementary Figure 2** Subgroup analysis of associations of residual-adjusted intake of beans with CKD. CKD, chronic kidney disease; OR, odds ratio; CI, confidence interval. The stratifications are (A) sex, (B) age, (C) ethnicity, (D) BMI, (E) hypertension, and (F) diabetes. The blue and horizontal bars represent the ORs and 95% CIs for bean. The Q1 group served as the reference group. ORs and 95% CIs were calculated using a multivariable logistic regression

analysis model with adjustment for age, sex, ethnicity, BMI, hypertension, diabetes, smoking status, drinking status, annual family income, education, physical activity, energy intake and dietary pattern (without beans and bean products). Stratification variables were omitted from the adjustment set in the corresponding stratified analyses.

**Supplementary Table 4** Sensitivity analysis of the association between residual-adjusted intake of beans and bean products, soy isoflavones and CKD.

**Supplementary Table 5** Comparison of baseline characteristics between excluded and included groups.

**Supplementary Table 1 Specific variables and proportion of missing data**

| Variables | Missing | Percentage of missing |
|---|---|---|
| Dietary intake | 873 | 2.40% |
| Height | 645 | 1.77% |
| Weight | 853 | 2.35% |
| Waist | 873 | 2.40% |
| Sex | 873 | 2.40% |
| Age | 916 | 2.52% |
| Ethnicity | 927 | 2.55% |
| Annual family income | 953 | 2.62% |
| Education | 883 | 2.43% |
| Current smoker | 876 | 2.41% |
| Current drinker | 878 | 2.41% |
| SBP/DBP | 942 | 2.59% |
| Proteinuria | 4488 | 12.34% |
| Creatinine | 770 | 2.12% |
| TC | 777 | 2.14% |
| TG | 777 | 2.14% |
| FBG | 777 | 2.14% |
| SUA | 2624 | 7.21% |
| Soy isoflavones and their subtypes | 873 | 2.40% |
| Energy intake | 873 | 2.40% |
| Protein intake | 873 | 2.40% |
| Fat intake | 873 | 2.40% |
| Carbohydrate intake | 873 | 2.40% |

SBP, systolic blood pressure; DBP, diastolic blood pressure; TC, total cholesterol; TG, triglycerides; FBG, fasting blood glucose; SUA, serum uric acid.

Missing data are presented as the number of participants with missing information and the corresponding percentage of the total study sample. The percentages were calculated using the overall sample of eligible participants (N = 36,376) as the denominator. Analyses in the main text were conducted on participants with complete data for all variables included in the respective models.

**Supplementary Table 2 Baseline characteristics of study participants stratified by Beans and bean products residual-adjusted intake**

| Variables | Total (N = 27,003) | Quartile of Beans and bean products residual-adjusted intake | | | | *P*-value |
|---|---|---|---|---|---|---|
| | | Q1 (n= 6750) | Q2 (n = 6751) | Q3 (n = 6751) | Q4 (n = 6751) | |
| **Age, years, n (%)** | | | | | | <0001 |
| <50 | 9296 (34.43) | 2561 (37.94) | 2214 (32.80) | 2100 (31.11) | 2421 (35.86) | |
| ≥50 | 17707 (65.57) | 4189 (62.06) | 4537 (67.20) | 4651 (68.89) | 4330 (64.14) | |
| **Sex, n (%)** | | | | | | <0001 |
| Men | 9472 (35.08) | 3278 (48.56) | 2119 (31.39) | 1800 (26.66) | 2275 (33.70) | |
| Women | 17531 (64.92) | 3472 (51.44) | 4632 (68.61) | 4951 (73.34) | 4476 (66.30) | |
| **Ethnicity, n (%)** | | | | | | <0001 |
| Han | 14581 (54.00) | 3370 (49.93) | 4105 (60.81) | 3973 (58.85) | 3133 (46.41) | |
| Other | 12422 (46.00) | 3380 (50.07) | 2646 (39.19) | 2778 (41.15) | 3618 (53.59) | |
| **Education, n (%)** | | | | | | <0001 |
| Middle school and below | 16188 (59.95) | 3653 (54.12) | 4311 (63.86) | 4406 (65.26) | 3818 (56.55) | |
| High school | 5452 (20.19) | 1465 (21.70) | 1276 (18.90) | 1268 (18.78) | 1443 (21.37) | |
| College and above | 5363 (19.86) | 1632 (24.18) | 1164 (17.24) | 1077 (15.95) | 1490 (22.07) | |
| **Annual family income, RMB, n (%)** | | | | | | <0001 |
| <50,000 | 11522 (42.67) | 2675 (39.63) | 3014 (44.65) | 3178 (47.07) | 2655 (39.33) | |
| 50,000-100,000 | 10983 (40.67) | 2668 (39.53) | 2655 (39.33) | 2610 (38.66) | 3050 (45.18) | |
| >100,000 | 4498 (16.66) | 1407 (20.84) | 1082 (16.03) | 963 (14.26) | 1046 (15.49) | |
| **Current smoker, n (%)** | 5862 (21.71) | 2055 (30.44) | 1374 (20.35) | 1121 (16.60) | 1312 (19.43) | <0001 |
| **Current drinker, n (%)** | 6073 (22.49) | 2450 (36.30) | 1260 (18.66) | 1070 (15.85) | 1293 (19.15) | <0001 |
| **Physical activity, MET-h/wk, n (%)** | | | | | | <0001 |
| ≤50 | 7379 (27.33) | 1736 (25.72) | 2019 (29.91) | 2061 (30.53) | 1563 (23.15) | |
| 51-100 | 8405 (31.13) | 1964 (29.10) | 2075 (30.74) | 2123 (31.45) | 2243 (33.22) | |
| 101-150 | 4816 (17.83) | 1337 (19.81) | 1152 (17.06) | 1111 (16.46) | 1216 (18.01) | |
| ＞150 | 6403 (23.71) | 1713 (25.38) | 1505 (22.29) | 1456 (21.57) | 1729 (25.61) | |
| **BMI, kg/m$^2$, n (%)** | | | | | | 0.297 |
| <24 | 9969 (36.92) | 2445 (36.22) | 2543 (37.67) | 2431 (36.01) | 2550 (37.77) | |

| | | | | | | |
|---|---|---|---|---|---|---|
| ≥24 | 17034 (63.08) | 4305 (63.78) | 4208 (62.33) | 4320 (63.99) | 4201 (62.23) | |
| **Waist, n (%)** | | | | | | 0.458 |
| Normal | 12862 (47.63) | 3233 (47.90) | 3217 (47.64) | 3162 (46.84) | 3250 (48.14) | |
| Obesity | 14141 (52.37) | 3517 (52.10) | 3534 (52.35) | 3589 (53.16) | 3501 (51.86) | |
| **SBP, mmHg, median, (IQR)** | 131.34 (119.67, 145.67) | 130.00 (118.33, 143.33) | 131.67 (119.67, 146.00) | 132.67 (120.33, 147.67) | 131.33 (120.00, 145.33) | <0001 |
| **DBP, mmHg, median, (IQR)** | 79.34 (72.00, 87.00) | 79.33 (71.67, 87.00) | 79.33 (72.33, 87.00) | 79.67 (72.33, 87.00) | 79.00 (72.00, 86.33) | 0.026 |
| **FBG, mmol/L, median, (IQR)** | 5.33 (4.91, 5.90) | 5.27 (4.84, 5.81) | 5.30 (4.90, 5.84) | 5.37 (4.97, 5.92) | 5.40 (5.00, 6.00) | <0001 |
| **SUA, µmol/L, median, (IQR)** | 302.00 (252.70, 364.20) | 312.55 (260.10, 378.00) | 298.70 (250.40, 359.70) | 296.00 (250.00, 353.60) | 302.10 (251.50, 365.40) | <0001 |
| **Hypertension, n (%)** | 11087 (41.06) | 2638 (39.08) | 2780 (41.18) | 2938 (43.52) | 2731 (40.45) | <0001 |
| **Diabetes, n (%)** | 3146 (11.65) | 678 (10.04) | 719 (10.65) | 833 (12.34) | 916 (13.57) | <0001 |
| **Hyperuricemia, n (%)** | 3120 (11.55) | 981 (14.53) | 720 (10.67) | 624 (9.24) | 795 (11.78) | <0001 |
| **Eenergy intake, kcal/d, median, (IQR)** | 1371.09 (1045.71, 1796.07) | 1704.89 (1413.40, 2061.11) | 1188.13 (976.64, 1469.16) | 1075.04 (858.87, 1423.97) | 1537.80 (1171.95, 1979.89) | <0001 |
| **Carbohydrate intake, g/d, median, (IQR)** | 209.81 (163.25, 269.46) | 259.31 (215.90, 311.41) | 191.20 (155.53, 230.42) | 170.91 (135.69, 218.74) | 221.71 (173.55, 284.90) | <0001 |
| **Protein intake, g/d, median, (IQR)** | 55.28 (42.00, 73.55) | 64.33 (52.48, 79.15) | 46.83 (37.99, 58.31) | 44.77 (35.47, 60.24) | 68.00 (51.84, 87.28) | <0001 |
| **Fat intake, g/d, median, (IQR)** | 32.92 (22.89, 46.88) | 40.59 (30.13, 54.01) | 26.56 (19.66, 36.38) | 25.61 (18.45, 36.48) | 41.38 (29.58, 55.36) | <0001 |
| **Meat intake, g/d, median, (IQR)** | 42.84 (17.16, 64.92) | 63.68 (30.00, 86.56) | 42.84 (17.16, 64.26) | 30.00 (13.50, 60.00) | 40.00 (17.16, 63.68) | <.0001 |
| **Fish intake, g/d, median, (IQR)** | 20.45 (9.84, 31.69) | 18.93 (8.20, 32.30) | 18.86 (7.38, 28.27) | 20.02 (9.84, 29.84) | 22.55 (13.94, 35.35) | <.0001 |
| **Vegetable intake, g/d, median, (IQR)** | 193.14 (123.57, 291.65) | 213.41 (139.37, 308.31) | 160.69 (109.27, 247.51) | 168.89 (110.57, 255.01) | 239.10 (155.54, 343.14) | <.0001 |
| **Fruit intake, g/d, median, (IQR)** | 115.62 (49.86, 224.90) | 145.76 (66.05, 275.78) | 101.99 (39.76, 198.19) | 83.32 (31.81, 169.17) | 140.24 (67.04, 254.72) | <.0001 |
| **CKD, n (%)** | 900 (3.33) | 286 (4.24) | 244 (3.61) | 193 (2.86) | 177 (2.62) | <.0001 |
| Urine protein, n (%) | 808 (2.99) | 261 (3.87) | 219 (3.24) | 169 (2.50) | 159 (2.36) | <.0001 |

| | | | | | | |
|---|---|---|---|---|---|---|
| eGFR <60 mL/min/1.73 m², n (%) | 157 (0.58) | 53 (0.79) | 45 (0.67) | 34 (0.50) | 25 (0.37) | 0.009 |
| Both, n (%) | 65 (0.24) | 28 (0.41) | 20 (0.30) | 10 (0.15) | 7 (0.10) | 0.001 |

CKD, chronic kidney disease; BMI, body mass index; MET, metabolic equivalent; IQR, interquartile range; SBP, systolic blood pressure; DBP, diastolic blood pressure; FBG, fasting blood glucose; SUA, serum uric acid.

Since none of the continuous variables followed a normal distribution, the Kruskal-Wallis test was used to compare differences across quartile groups, and variables were described using medians (25th percentile, 75th percentile). For categorical variables, chi-square tests were employed to calculate P-values, and distributions were presented as counts (percentages). Intake of total beans and bean products was adjusted for energy intake using the residual method. The resulting values represent relative intake levels independent of total energy. Residual-adjusted intake was categorized into quartile groups (Q1, Q2, Q3, and Q4) using the following cutoff points: 44.77, 68.48, and 100.92 g/d.

**Supplementary Table 3 Subgroup analysis of the association between Soy isoflavones residual-adjusted intake and CKD**

| Subgroup | CKD/without CKD | OR (95%CI) | *P* for trend | *P* for interaction |
|---|---|---|---|---|
| **Sex** | | | | 0.011 |
| Men | | | 0.056 | |
| Q1 | 168/3068 | 1.00 (reference) | | |
| Q2 | 81/1978 | 0.74 (0.55, 0.98) | | |
| Q3 | 75/1782 | 0.75 (0.56, 1.01) | | |
| Q4 | 95/2225 | 0.76 (0.58, 0.99) | | |
| Women | | | <0.001 | |
| Q1 | 118/3396 | 1.00 (reference) | | |
| Q2 | 141/4551 | 0.89 (0.68, 1.17) | | |
| Q3 | 126/4768 | 0.74 (0.55, 0.96) | | |
| Q4 | 96/4335 | 0.66 (0.50, 0.89) | | |
| **Age, years** | | | | 0.192 |
| < 50 | | | 0.031 | |
| Q1 | 91/2542 | 1.00 (reference) | | |
| Q2 | 67/2239 | 1.01 (0.71, 1.43) | | |
| Q3 | 53/2051 | 0.86 (0.59, 1.25) | | |
| Q4 | 47/2206 | 0.70 (0.48, 1.01) | | |
| ≥ 50 | | | 0.005 | |
| Q1 | 195/3922 | 1.00 (reference) | | |
| Q2 | 155/4290 | 0.76 (0.60, 0.96) | | |
| Q3 | 148/4499 | 0.69 (0.54, 0.88) | | |
| Q4 | 144/4354 | 0.70 (0.55, 0.88) | | |
| **Ethnicity** | | | | 0.031 |
| Han | | | 0.600 | |
| Q1 | 85/3154 | 1.00 (reference) | | |
| Q2 | 103/3850 | 0.84 (0.61, 1.16) | | |
| Q3 | 110/4015 | 0.79 (0.57, 1.09) | | |
| Q4 | 86/3178 | 0.89 (0.65, 1.23) | | |
| Other | | | <0.001 | |
| Q1 | 201/3310 | 1.00 (reference) | | |
| Q2 | 119/2679 | 0.85 (0.66, 1.09) | | |
| Q3 | 91/2535 | 0.72 (0.55, 0.94) | | |
| Q4 | 105/3382 | 0.65 (0.50, 0.84) | | |
| **BMI, kg/m2** | | | | 0.149 |
| < 24 | | | 0.021 | |
| Q1 | 70/2411 | 1.00 (reference) | | |
| Q2 | 50/2482 | 0.69 (0.46, 1.02) | | |
| Q3 | 49/2386 | 0.66 (0.44, 0.99) | | |
| Q4 | 46/2475 | 0.60 (0.40, 0.89) | | |
| ≥ 24 | | | 0.007 | |

| | | | | |
|---|---|---|---|---|
| Q1 | 216/4053 | 1.00 (reference) | | |
| Q2 | 172/4047 | 0.88 (0.70, 1.10) | | |
| Q3 | 152/4164 | 0.76 (0.60, 0.96) | | |
| Q4 | 145/4085 | 0.74 (0.59, 0.93) | | |
| **Hypertension** | | | | 0.841 |
| Yes | | | 0.019 | |
| Q1 | 178/2417 | 1.00 (reference) | | |
| Q2 | 144/2582 | 0.83 (0.65, 1.06) | | |
| Q3 | 138/2811 | 0.74 (0.57, 0.95) | | |
| Q4 | 136/2681 | 0.74 (0.58, 0.94) | | |
| No | | | 0.008 | |
| Q1 | 108/4047 | 1.00 (reference) | | |
| Q2 | 78/3947 | 0.84 (0.61, 1.15) | | |
| Q3 | 63/3739 | 0.75 (0.53, 1.06) | | |
| Q4 | 55/3879 | 0.64 (0.45, 0.90) | | |
| **Diabetes** | | | | 0.033 |
| Yes | | | 0.014 | |
| Q1 | 75/591 | 1.00 (reference) | | |
| Q2 | 61/669 | 0.67 (0.45, 0.99) | | |
| Q3 | 58/775 | 0.54 (0.36, 0.80) | | |
| Q4 | 63/854 | 0.59 (0.40, 0.86) | | |
| No | | | 0.012 | |
| Q1 | 211/5873 | 1.00 (reference) | | |
| Q2 | 161/5860 | 0.89 (0.71, 1.12) | | |
| Q3 | 143/5775 | 0.83 (0.65, 1.05) | | |
| Q4 | 128/5706 | 0.75 (0.59, 0.94) | | |

CKD, chronic kidney disease; OR, odds ratio; CI, confidence interval; BMI, body mass index. Multivariable logistic regression models were adjusted for age, sex, ethnicity, BMI, hypertension, diabetes, smoking status, drinking status, annual family income, education, physical activity, energy intake, and dietary pattern (derived without beans and bean products). Stratification variables were omitted from the adjustment set in the corresponding stratified analyses. The lowest quartile (Q1) of residual-adjusted soy isoflavone intake was used as the reference group. *P* for trend was calculated by assigning the median value of each quartile to the respective category and treating it as a continuous variable. *P* for interaction was obtained from the likelihood ratio test comparing models with and without the product term between soy isoflavone quartile and the stratification variable.

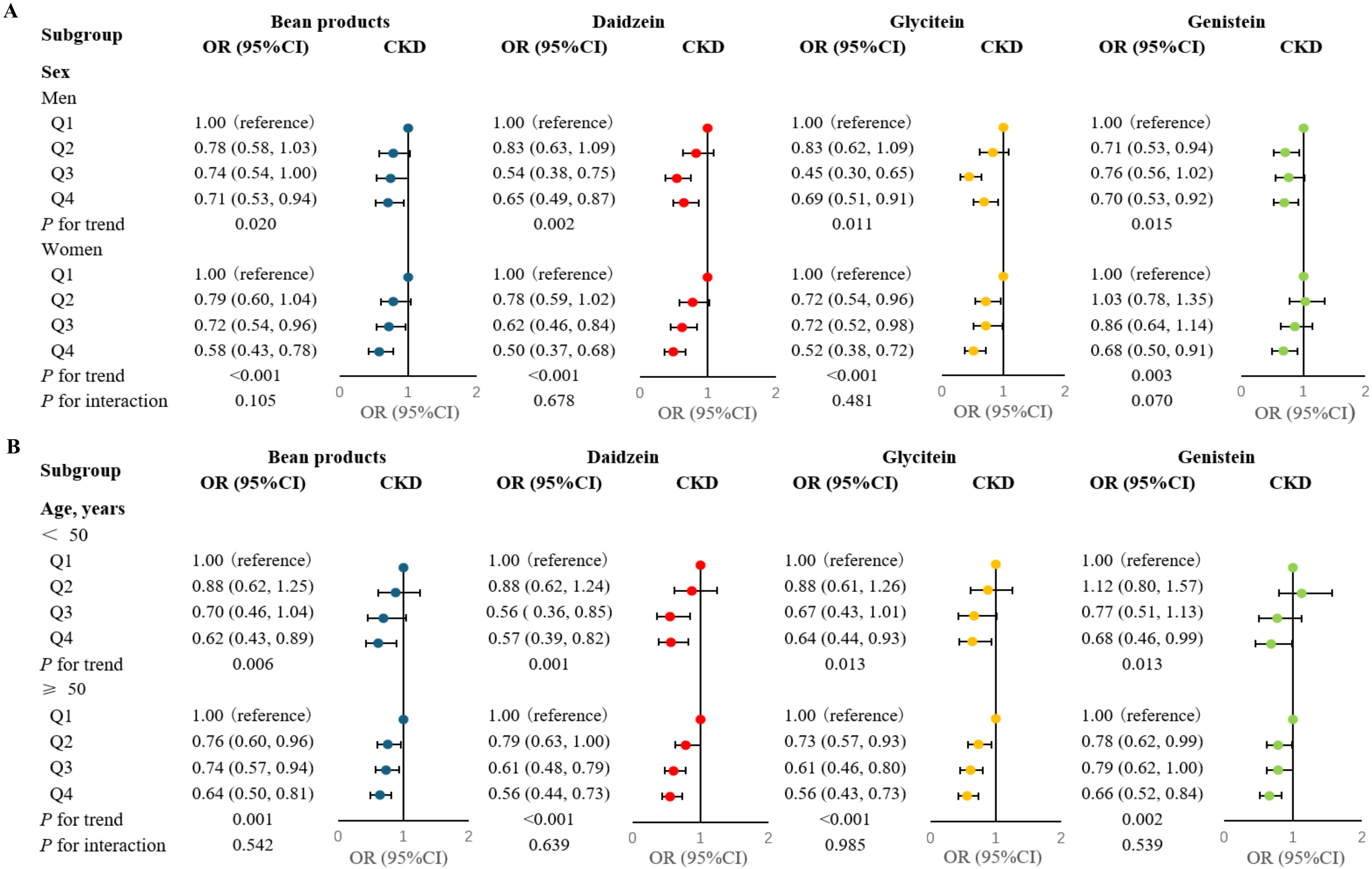

A
Subgroup
Bean products
Daidzein
Glycitein
Genistein
OR (95%CI) CKD OR (95%CI) CKD OR (95%CI) CKD OR (95%CI) CKD
Sex
Men
Q1 1.00（reference） 1.00（reference） 1.00（reference） 1.00（reference）
Q2 0.78 (0.58, 1.03) 0.83 (0.63, 1.09) 0.83 (0.62, 1.09) 0.71 (0.53, 0.94)
Q3 0.74 (0.54, 1.00) 0.54 (0.38, 0.75) 0.45 (0.30, 0.65) 0.76 (0.56, 1.02)
Q4 0.71 (0.53, 0.94) 0.65 (0.49, 0.87) 0.69 (0.51, 0.91) 0.70 (0.53, 0.92)
P for trend 0.020 0.002 0.011 0.015
Women
Q1 1.00（reference） 1.00（reference） 1.00（reference） 1.00（reference）
Q2 0.79 (0.60, 1.04) 0.78 (0.59, 1.02) 0.72 (0.54, 0.96) 1.03 (0.78, 1.35)
Q3 0.72 (0.54, 0.96) 0.62 (0.46, 0.84) 0.72 (0.52, 0.98) 0.86 (0.64, 1.14)
Q4 0.58 (0.43, 0.78) 0.50 (0.37, 0.68) 0.52 (0.38, 0.72) 0.68 (0.50, 0.91)
P for trend <0.001 <0.001 <0.001 0.003
P for interaction 0.105 0.678 0.481 0.070
0 1 2
OR (95%CI)
B
Subgroup
Bean products
Daidzein
Glycitein
Genistein
OR (95%CI) CKD OR (95%CI) CKD OR (95%CI) CKD OR (95%CI) CKD
Age, years
＜ 50
Q1 1.00（reference） 1.00（reference） 1.00（reference） 1.00（reference）
Q2 0.88 (0.62, 1.25) 0.88 (0.62, 1.24) 0.88 (0.61, 1.26) 1.12 (0.80, 1.57)
Q3 0.70 (0.46, 1.04) 0.56 ( 0.36, 0.85) 0.67 (0.43, 1.01) 0.77 (0.51, 1.13)
Q4 0.62 (0.43, 0.89) 0.57 (0.39, 0.82) 0.64 (0.44, 0.93) 0.68 (0.46, 0.99)
P for trend 0.006 0.001 0.013 0.013
⩾ 50
Q1 1.00（reference） 1.00（reference） 1.00（reference） 1.00（reference）
Q2 0.76 (0.60, 0.96) 0.79 (0.63, 1.00) 0.73 (0.57, 0.93) 0.78 (0.62, 0.99)
Q3 0.74 (0.57, 0.94) 0.61 (0.48, 0.79) 0.61 (0.46, 0.80) 0.79 (0.62, 1.00)
Q4 0.64 (0.50, 0.81) 0.56 (0.44, 0.73) 0.56 (0.43, 0.73) 0.66 (0.52, 0.84)
P for trend 0.001 <0.001 <0.001 0.002
P for interaction 0.542 0.639 0.985 0.539
0 1 2
OR (95%CI)

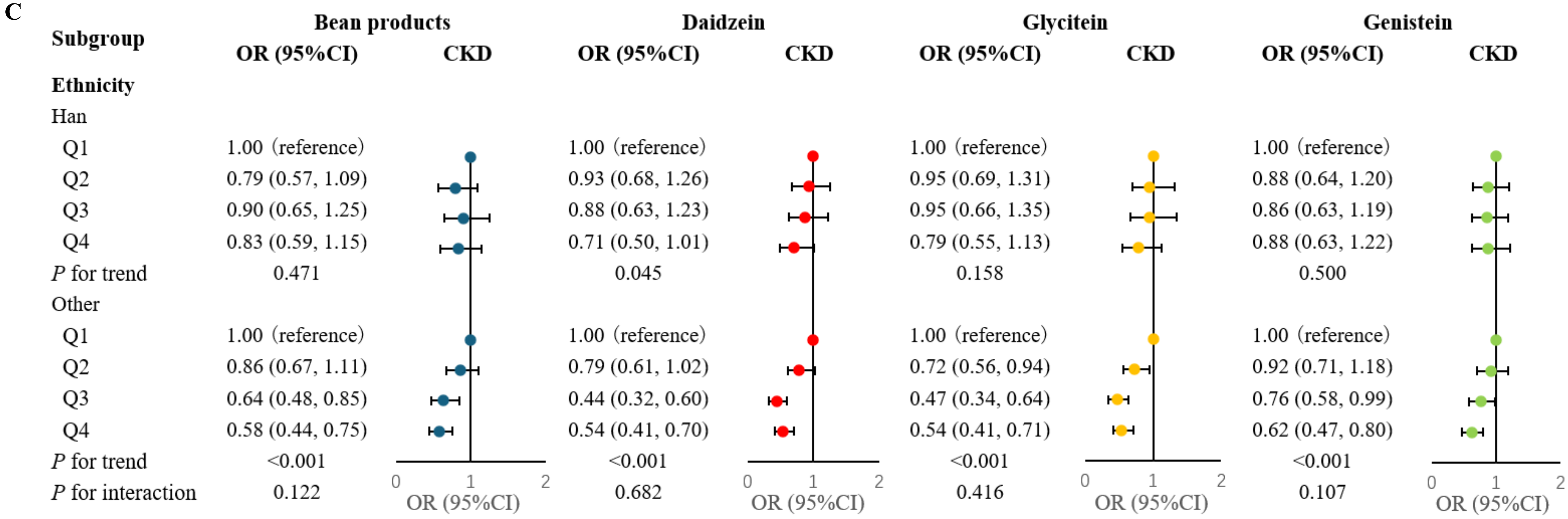
C
Subgroup
Bean products
Daidzein
Glycitein
Genistein
OR (95%CI) CKD
Ethnicity
Han
Q1 1.00（reference） 1.00（reference） 1.00（reference） 1.00（reference）
Q2 0.79 (0.57, 1.09) 0.93 (0.68, 1.26) 0.95 (0.69, 1.31) 0.88 (0.64, 1.20)
Q3 0.90 (0.65, 1.25) 0.88 (0.63, 1.23) 0.95 (0.66, 1.35) 0.86 (0.63, 1.19)
Q4 0.83 (0.59, 1.15) 0.71 (0.50, 1.01) 0.79 (0.55, 1.13) 0.88 (0.63, 1.22)
P for trend 0.471 0.045 0.158 0.500
Other
Q1 1.00（reference） 1.00（reference） 1.00（reference） 1.00（reference）
Q2 0.86 (0.67, 1.11) 0.79 (0.61, 1.02) 0.72 (0.56, 0.94) 0.92 (0.71, 1.18)
Q3 0.64 (0.48, 0.85) 0.44 (0.32, 0.60) 0.47 (0.34, 0.64) 0.76 (0.58, 0.99)
Q4 0.58 (0.44, 0.75) 0.54 (0.41, 0.70) 0.54 (0.41, 0.71) 0.62 (0.47, 0.80)
P for trend <0.001 <0.001 <0.001 <0.001
P for interaction 0.122 0.682 0.416 0.107
0 1 2
OR (95%CI)

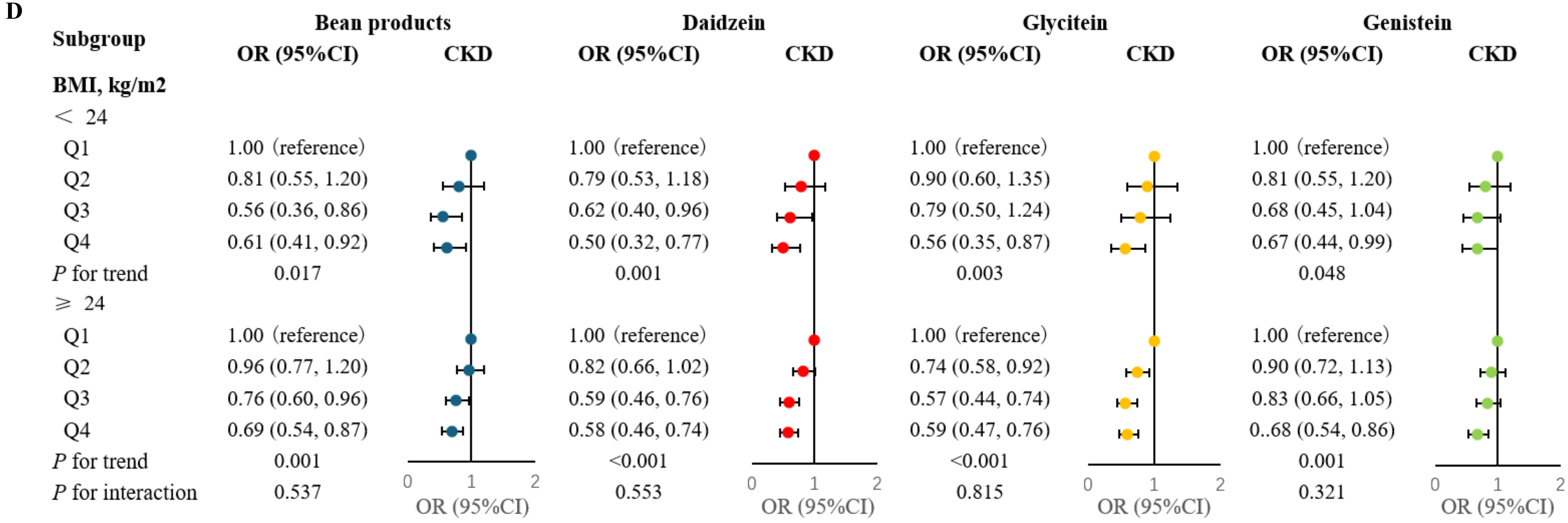
D
Subgroup
Bean products
Daidzein
Glycitein
Genistein
OR (95%CI) CKD
BMI, kg/m2
＜ 24
Q1 1.00（reference） 1.00（reference） 1.00（reference） 1.00（reference）
Q2 0.81 (0.55, 1.20) 0.79 (0.53, 1.18) 0.90 (0.60, 1.35) 0.81 (0.55, 1.20)
Q3 0.56 (0.36, 0.86) 0.62 (0.40, 0.96) 0.79 (0.50, 1.24) 0.68 (0.45, 1.04)
Q4 0.61 (0.41, 0.92) 0.50 (0.32, 0.77) 0.56 (0.35, 0.87) 0.67 (0.44, 0.99)
P for trend 0.017 0.001 0.003 0.048
⩾ 24
Q1 1.00（reference） 1.00（reference） 1.00（reference） 1.00（reference）
Q2 0.96 (0.77, 1.20) 0.82 (0.66, 1.02) 0.74 (0.58, 0.92) 0.90 (0.72, 1.13)
Q3 0.76 (0.60, 0.96) 0.59 (0.46, 0.76) 0.57 (0.44, 0.74) 0.83 (0.66, 1.05)
Q4 0.69 (0.54, 0.87) 0.58 (0.46, 0.74) 0.59 (0.47, 0.76) 0..68 (0.54, 0.86)
P for trend 0.001 <0.001 <0.001 0.001
P for interaction 0.537 0.553 0.815 0.321
0 1 2
OR (95%CI)

**E**

| Subgroup | Bean products OR (95%CI) | Daidzein OR (95%CI) | Glycitein OR (95%CI) | Genistein OR (95%CI) |
|---|---|---|---|---|
| **Hypertension** | | | | |
| Yes | | | | |
| Q1 | 1.00 (reference) | 1.00 (reference) | 1.00 (reference) | 1.00 (reference) |
| Q2 | 0.98 (0.77, 1.25) | 0.80 (0.63, 1.02) | 0.79 (0.61, 1.01) | 0.89 (0.70, 1.14) |
| Q3 | 0.80 (0.62, 1.03) | 0.66 (0.51, 0.87) | 0.63 (0.47, 0.84) | 0.79 (0.61, 1.01) |
| Q4 | 0.69 (0.53, 0.89) | 0.58 (0.45, 0.76) | 0.63 (0.48, 0.81) | 0.72 (0.56, 0.92) |
| *P* for trend | 0.002 | <0.001 | 0.002 | 0.007 |
| No | | | | |
| Q1 | 1.00 (reference) | 1.00 (reference) | 1.00 (reference) | 1.00 (reference) |
| Q2 | 0.83 (0.60, 1.14) | 0.84 (0.61, 1.15) | 0.77 (0.55, 1.07) | 0.85 (0.62, 1.17) |
| Q3 | 0.56 (0.38, 0.80) | 0.49 (0.33, 0.71) | 0.64 (0.44, 0.94) | 0.82 (0.58, 1.15) |
| Q4 | 0.65 (0.46, 0.90) | 0.54 (0.38, 0.77) | 0.53 (0.37, 0.76) | 0.60 (0.42, 0.85) |
| *P* for trend | 0.006 | <0.001 | 0.001 | 0.004 |
| *P* for interaction | 0.927 | 0.544 | 0.828 | 0.812 |

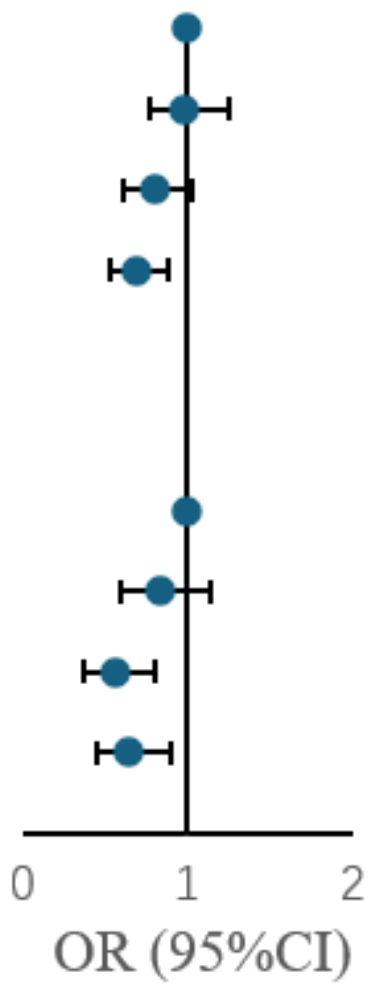

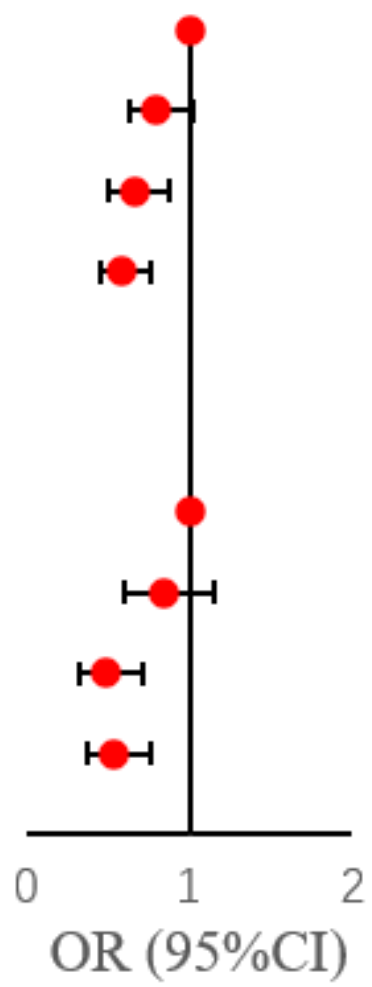

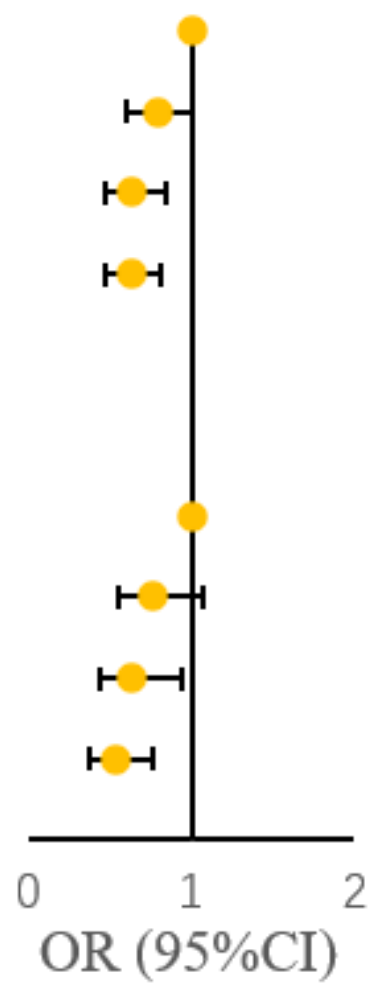

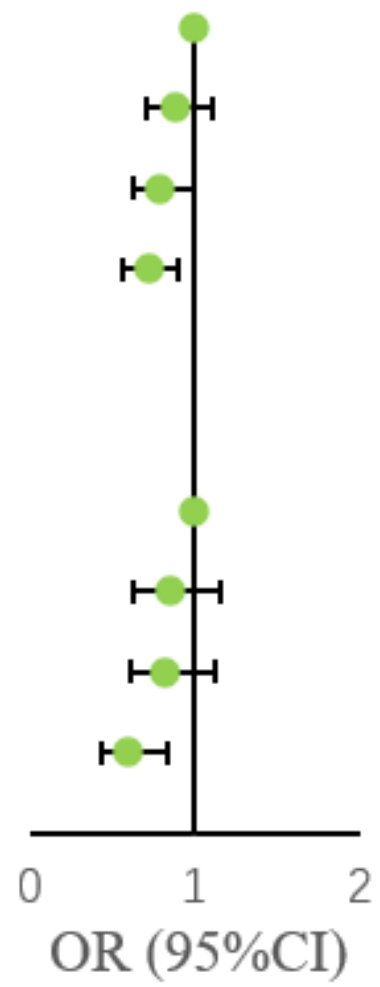


**F**

| Subgroup | Bean products OR (95%CI) | Daidzein OR (95%CI) | Glycitein OR (95%CI) | Genistein OR (95%CI) |
|---|---|---|---|---|
| **Diabetes** | | | | |
| Yes | | | | |
| Q1 | 1.00 (reference) | 1.00 (reference) | 1.00 (reference) | 1.00 (reference) |
| Q2 | 0.82 (0.55,1.21) | 0.81 (0.55, 1.19) | 0.91 (0.61, 1.35) | 0.79 (0.54, 1.16) |
| Q3 | 0.65 (0.43, 0.97) | 0.55 (0.36, 0.84) | 0.62 (0.40, 0.97) | 0.56 (0.37, 0.84) |
| Q4 | 0.51 (0.34, 0.75) | 0.44 (0.29, 0.67) | 0.48 (0.31, 0.74) | 0.59 (0.40, 0.86) |
| *P* for trend | 0.001 | <0.001 | 0.001 | 0.006 |
| No | | | | |
| Q1 | 1.00 (reference) | 1.00 (reference) | 1.00 (reference) | 1.00 (reference) |
| Q2 | 0.96 (0.77, 1.20) | 0.80 (0.64, 1.00) | 0.72 (0.57, 0.91) | 0.91 (0.72, 1.14) |
| Q3 | 0.73 (0.57, 0.93) | 0.61 (0.47, 0.79) | 0.63 (0.48, 0.81) | 0.90 (0.71, 1.13) |
| Q4 | 0.75 (0.59, 0.95) | 0.62 (0.48, 0.78) | 0.63 (0.50, 0.80) | 0.71 (0.56, 0.90) |
| *P* for trend | 0.006 | <0.001 | 0.001 | 0.004 |
| *P* for interaction | 0.059 | 0.125 | 0.098 | 0.104 |

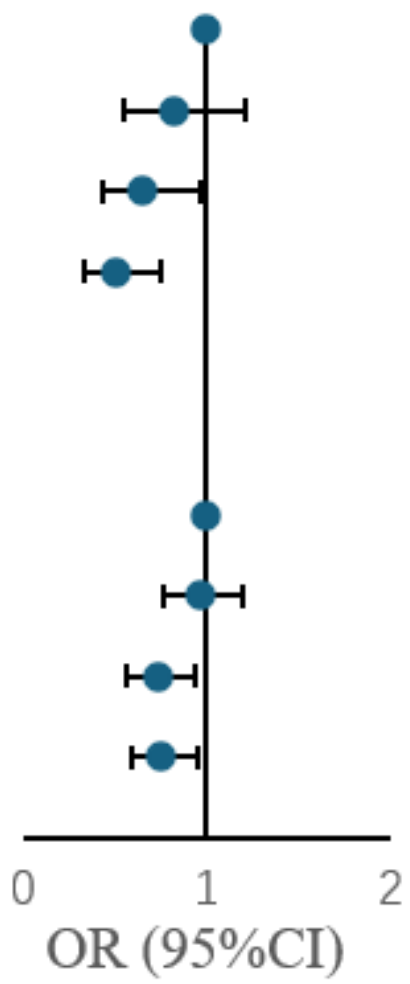

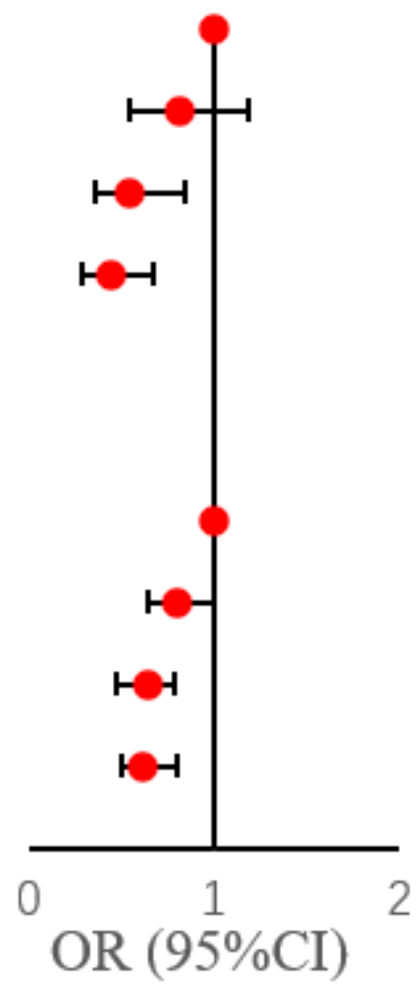

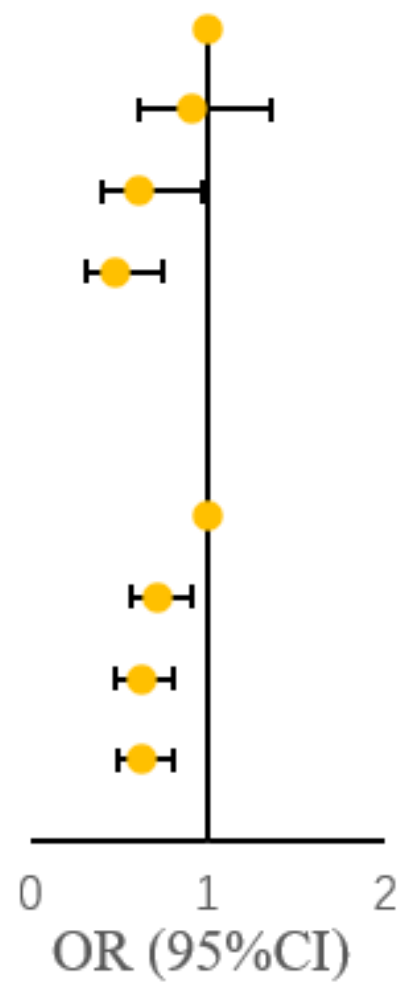

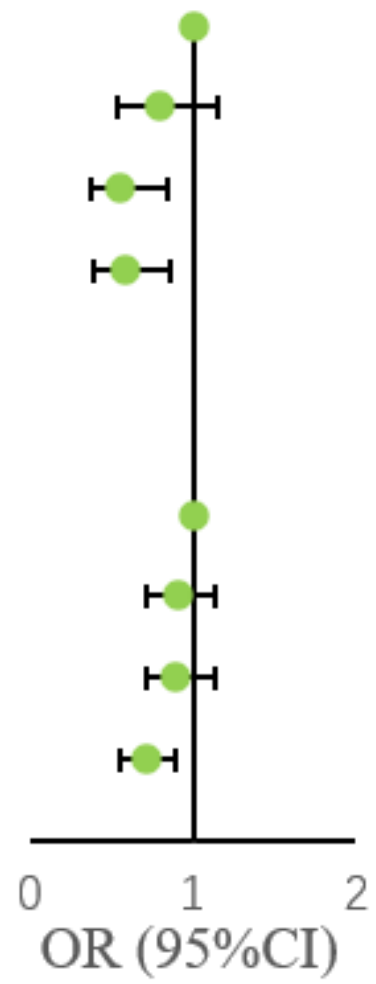

**Supplementary Figure 1** Subgroup analysis of associations of residual-adjusted intake of bean products, daidzein, glycitein, genistein with CKD. CKD, chronic kidney disease; OR, odds ratio; CI, confidence interval. The stratifications are (A) sex, (B) age, (C) ethnicity, (D) BMI, (E) hypertension, and (F) diabetes. The blue, red, yellow, and green dots and horizontal bars represent the ORs and 95% CIs for bean products, daidzein, glycitein, and genistein, respectively. The Q1 group served as the reference group. ORs and 95% CIs were calculated using a multivariable multivariate logistic regression analysis model with adjustment for age, sex, ethnicity, BMI, hypertension, diabetes, smoking status, drinking status, annual family income, education, physical activity, energy intake and dietary pattern (without beans and bean products). Stratification variables were omitted from the adjustment set in the corresponding stratified analyses.

A

| Subgroup | OR (95%CI) |
|---|---|
| **Sex** | |
| Men | |
| Q1 | 1.00（reference) |
| Q2 | 0.97 (0.73, 1.28) |
| Q3 | 0.83 (0.61, 1.11) |
| Q4 | 0.81 (0.61, 1.07) |
| *P* for trend | 0.110 |
| Women | |
| Q1 | 1.00 (reference) |
| Q2 | 0.99 (0.74, 1.33) |
| Q3 | 1.06 (0.80, 1.43) |
| Q4 | 1.08 (0.81, 1.45) |
| *P* for trend | 0.475 |
| *P* for interaction | 0.988 |

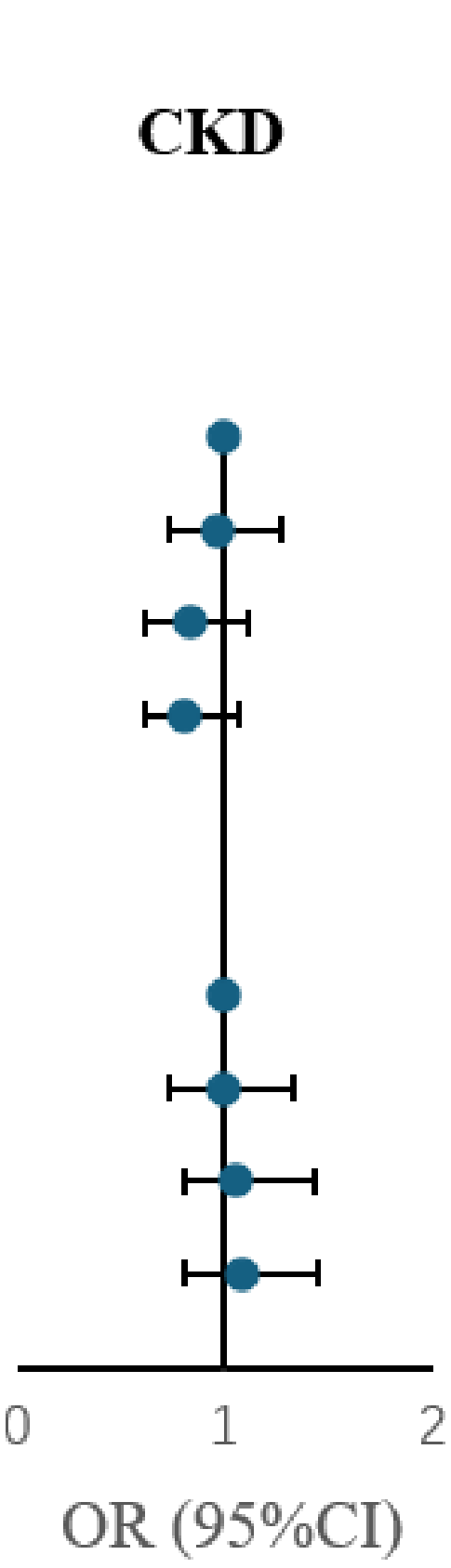


B

| Subgroup | OR (95%CI) |
|---|---|
| **Age, years** | |
| ＜ 50 | |
| Q1 | 1.00（reference) |
| Q2 | 0.92 (0.64, 1.34) |
| Q3 | 0.98 (0.67, 1.42) |
| Q4 | 0.90 (0.62, 1.30) |
| *P* for trend | 0.573 |
| ⩾ 50 | |
| Q1 | 1.00（reference) |
| Q2 | 0.97 (0.76, 1.24) |
| Q3 | 0.93 (0.73, 1.19) |
| Q4 | 0.95 (0.75, 1.20) |
| *P* for trend | 0.676 |
| *P* for interaction | 0.963 |

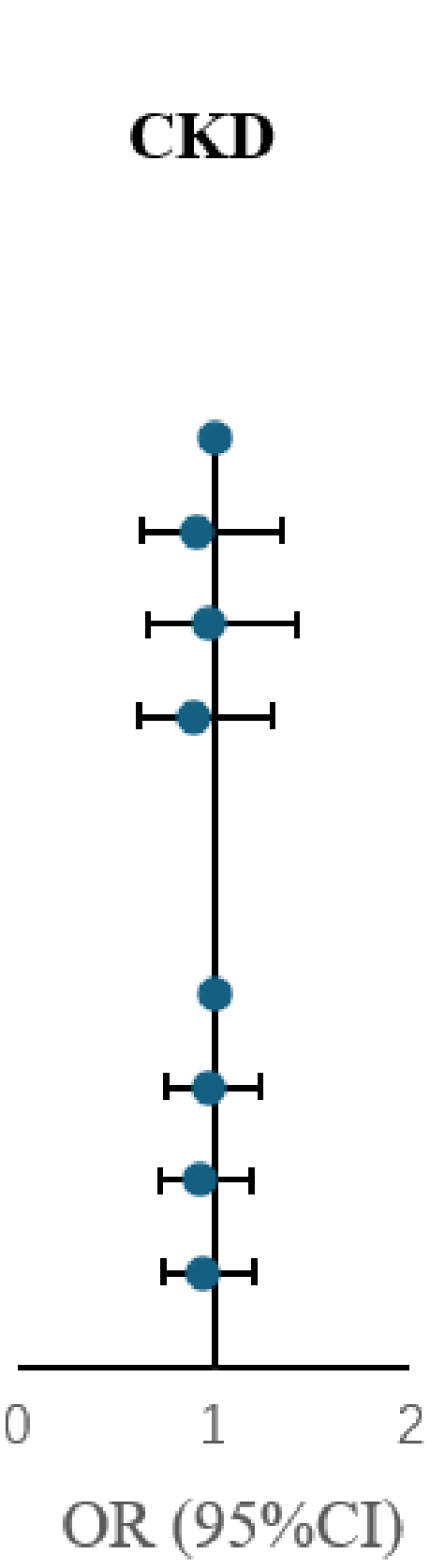

C

**Beans**

| **Subgroup** | **OR (95%CI)** |
|---|---|
| **Ethnicity** | |
| Han | |
| Q1 | 1.00（reference） |
| Q2 | 0.94 (0.70, 1.26) |
| Q3 | 0.76 (0.56, 1.05) |
| Q4 | 0.75 (0.54, 1.05) |
| *P* for trend | 0.060 |
| Other | |
| Q1 | 1.00（reference） |
| Q2 | 0.90 (0.68, 1.21) |
| Q3 | 1.04 (0.79, 1.36) |
| Q4 | 1.08 (0.84, 1.40) |
| *P* for trend | 0.332 |
| *P* for interaction | 0.978 |

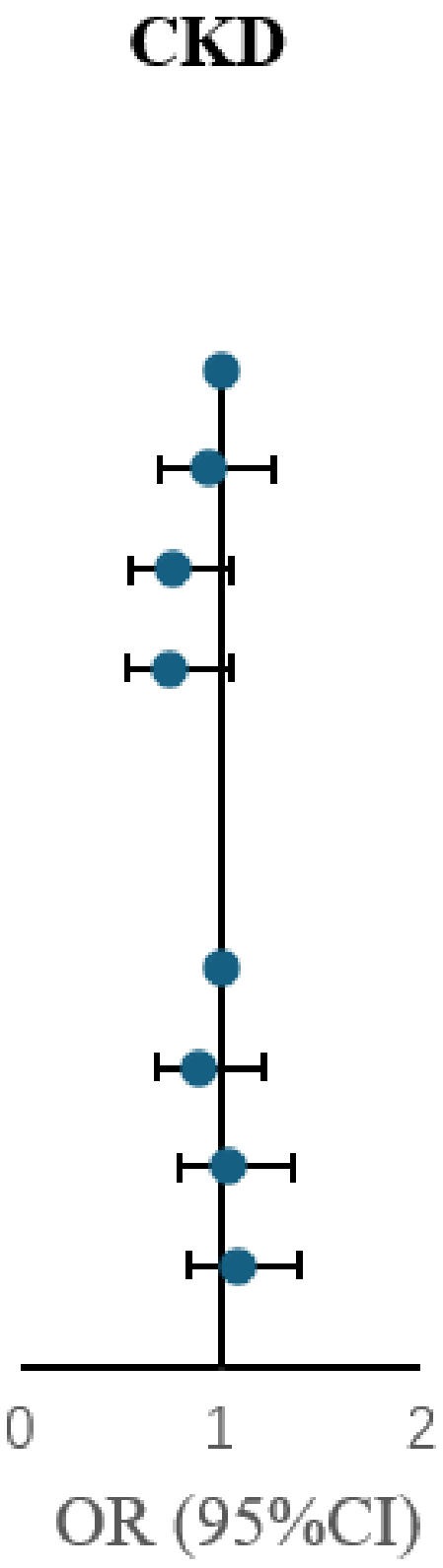


D

**Beans**

| **Subgroup** | **OR (95%CI)** |
|---|---|
| **BMI, kg/m2** | |
| ＜ 24 | |
| Q1 | 1.00（reference） |
| Q2 | 0.81 (0.54, 1.23) |
| Q3 | 0.90 (0.60, 1.34) |
| Q4 | 0.82 (0.55, 1.23) |
| *P* for trend | 0.459 |
| ⩾ 24 | |
| Q1 | 1.00（reference） |
| Q2 | 1.00 (0.80, 1.27) |
| Q3 | 0.94 (0.74, 1.19) |
| Q4 | 0.96 (0.77, 1.21) |
| *P* for trend | 0.705 |
| *P* for interaction | 0.634 |

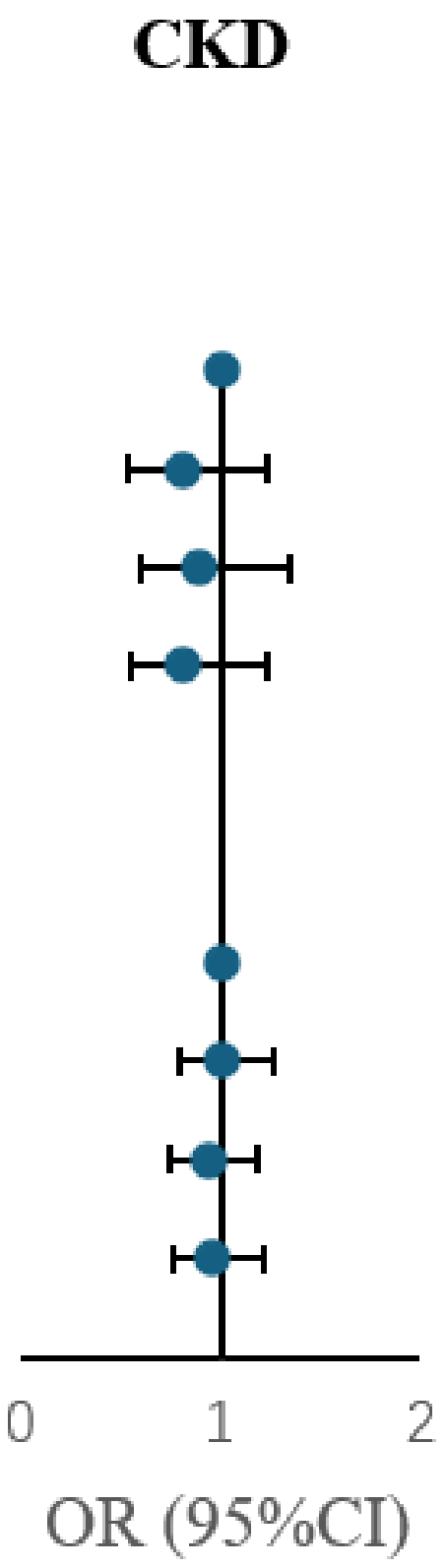

**E**

**Beans**

| **Subgroup** | **OR (95%CI)** |
|---|---|
| **Hypertension** | |
| Yes | |
| Q1 | 1.00（reference） |
| Q2 | 1.04 (0.81, 1.34) |
| Q3 | 0.97 (0.75, 1.26) |
| Q4 | 1.00 (0.78, 1.28) |
| *P* for trend | 0.893 |
| No | |
| Q1 | 1.00（reference） |
| Q2 | 0.84 (0.60, 1.17) |
| Q3 | 0.89 (0.63, 1.24) |
| Q4 | 0.82 (0.59, 1.14) |
| *P* for trend | 0.348 |
| *P* for interaction | 0.866 |

**CKD**

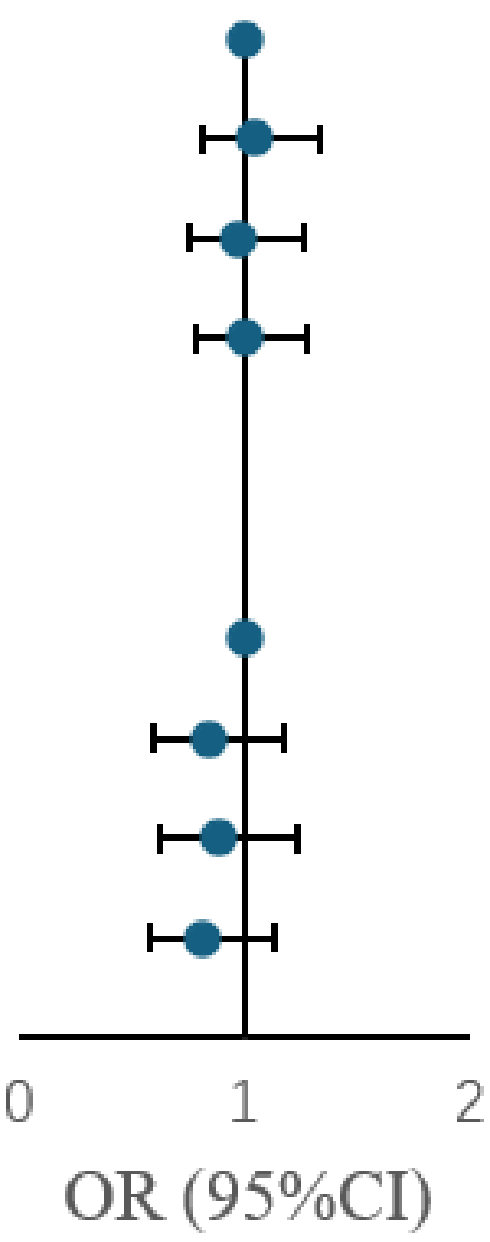


**F**

**Beans**

| **Subgroup** | **OR (95%CI)** |
|---|---|
| **Diabetes** | |
| Yes | |
| Q1 | 1.00（reference） |
| Q2 | 1.14 (0.76, 1.72) |
| Q3 | 0.90 (0.59, 1.37) |
| Q4 | 1.03 (0.69, 1.53) |
| *P* for trend | 0.904 |
| No | |
| Q1 | 1.00（reference） |
| Q2 | 0.89 (0.70, 1.13) |
| Q3 | 0.94 (0.74, 1.19) |
| Q4 | 0.90 (0.71, 1.13) |
| *P* for trend | 0.480 |
| *P* for interaction | 0.998 |

**CKD**

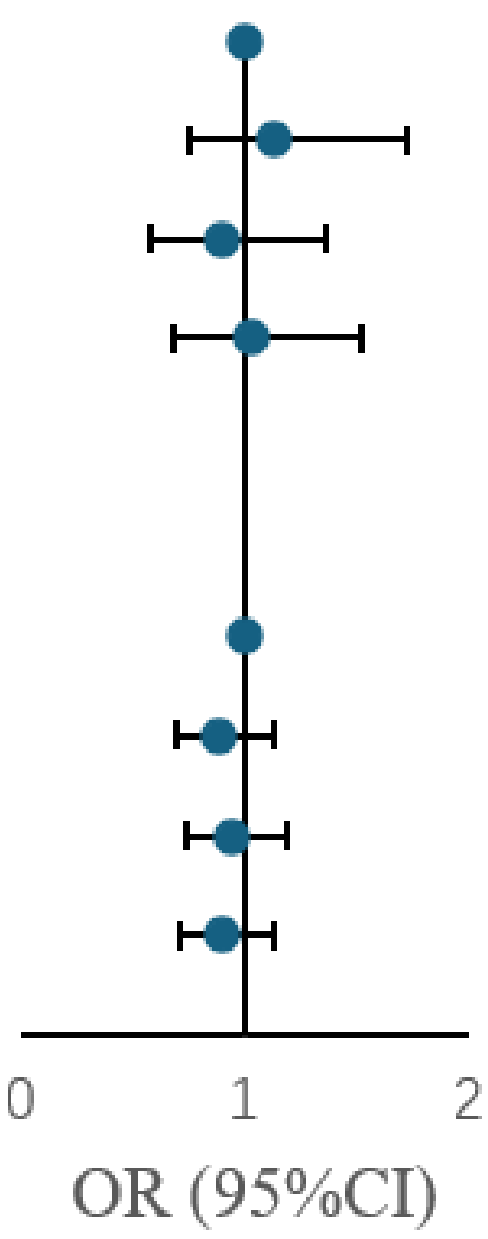


**Supplementary Figure 2** Subgroup analysis of associations of residual-adjusted intake of beans with CKD. CKD, chronic kidney disease; OR, odds ratio; CI, confidence

interval. The stratifications are (A) sex, (B) age, (C) ethnicity, (D) BMI, (E) hypertension, and (F) diabetes. The blue and horizontal bars represent the ORs and 95% CIs for bean. The Q1 group served as the reference group. ORs and 95% CIs were calculated using a multivariable logistic regression analysis model with adjustment for age, sex, ethnicity, BMI, hypertension, diabetes, smoking status, drinking status, annual family income, education, physical activity, energy intake and dietary pattern (without beans and bean products). Stratification variables were omitted from the adjustment set in the corresponding stratified analyses.

**Supplementary Table 4 Sensitivity analysis of the association between residual-adjusted intake of beans and bean products, soy isoflavones and CKD**

| Variables | Cases/Participants | Residual-adjusted intake | | | |
|---|---|---|---|---|---|
| | | Q1 | Q2 | Q3 | Q4 |
| **Total beans and bean products** | | | | | |
| Main analysis | 900/27003 | 1.00 (reference) | 0.92 (0.76, 1.12) | 0.71 (0.57, 0.87) | 0.67 (0.54, 0.82) |
| Sensitivity analysis 1[1] | 802/22074 | 1.00 (reference) | 0.91 (0.74, 1.12) | 0.70 (0.56, 0.88) | 0.66 (0.53, 0.82) |
| Sensitivity analysis 2[2] | 856/24827 | 1.00 (reference) | 0.93 (0.76, 1.13) | 0.70 (0.56, 0.87) | 0.64 (0.52, 0.79) |
| Sensitivity analysis 3[3] | 900/27003 | 1.00 (reference) | 0.91 (0.75, 1.10) | 0.70 (0.57, 0.87) | 0.66 (0.54, 0.81) |
| Sensitivity analysis 4[4] | 2449/27003 | 1.00 (reference) | 0.91 (0.81, 1.03) | 0.73 (0.64, 0.83) | 0.67 (0.59, 0.76) |
| Sensitivity analysis 5[5] | 900/27003 | 1.00 (reference) | 0.96 (0.79, 1.17) | 0.76 (0.61, 0.95) | 0.76 (0.59, 0.98) |
| Sensitivity analysis 6[6] | 808/26103 | 1.00 (reference) | 0.92 (0.75, 1.12) | 0.69 (0.55, 0.86) | 0.72 (0.60, 0.86) |
| Sensitivity analysis 7[7] | 157/26103 | 1.00 (reference) | 0.90 (0.58, 1.39) | 0.66 (0.40, 1.05) | 0.47 (0.28, 0.77) |
| **Beans** | | | | | |
| Main analysis | 900/27003 | 1.00 (reference) | 0.96 (0.78, 1.17) | 0.93 (0.76, 1.15) | 0.93 (0.76, 1.14) |
| Sensitivity analysis 1 | 802/22074 | 1.00 (reference) | 0.95 (0.77, 1.18) | 0.95 (0.76, 1.18) | 0.91 (0.73, 1.13) |
| Sensitivity analysis 2 | 856/24827 | 1.00 (reference) | 0.98 (0.79, 1.20) | 0.97 (0.78, 1.19) | 0.92 (0.75, 1.13) |
| Sensitivity analysis 3 | 900/27003 | 1.00 (reference) | 0.96 (0.78, 1.17) | 0.94 (0.76, 1.15) | 0.94 (0.77, 1.14) |
| Sensitivity analysis 4 | 2449/27003 | 1.00 (reference) | 0.92 (0.81, 1.04) | 0.89 (0.78, 1.01) | 0.96 (0.85, 1.09) |
| Sensitivity analysis 5 | 900/27003 | 1.00 (reference) | 0.99 (0.81, 1.21) | 0.99 (0.80, 1.22) | 1.03 (0.84, 1.26) |
| Sensitivity analysis 6 | 808/26103 | 1.00 (reference) | 0.98 (0.80, 1.22) | 0.96 (0.77, 1.19) | 0.93 (0.75, 1.15) |
| Sensitivity analysis 7 | 157/26103 | 1.00 (reference) | 0.68 (0.41, 1.10) | 0.64 (0.39, 1.03) | 0.98 (0.64, 1.51) |
| **Bean products** | | | | | |
| Main analysis | 900/27003 | 1.00 (reference) | 0.79 (0.65, 0.96) | 0.72 (0.59, 0.89) | 0.63 (0.51, 0.77) |
| Sensitivity analysis 1 | 802/22074 | 1.00 (reference) | 0.79 (0.64, 0.98) | 0.71 (0.56, 0.88) | 0.63 (0.51, 0.79) |
| Sensitivity analysis 2 | 856/24827 | 1.00 (reference) | 0.80 (0.65, 0.97) | 0.71 (0.57, 0.88) | 0.61 (0.49, 0.75) |
| Sensitivity analysis 3 | 900/27003 | 1.00 (reference) | 0.78 (0.64, 0.95) | 0.72 (0.58, 0.89) | 0.62 (0.51, 0.76) |

| | | | | | |
|---|---|---|---|---|---|
| Sensitivity analysis 4 | 2449/27003 | 1.00 (reference) | 0.88 (0.78, 1.00) | 0.68 (0.60, 0.78) | 0.65 (0.57, 0.73) |
| Sensitivity analysis 5 | 900/27003 | 1.00 (reference) | 0.82 (0.67, 1.00) | 0.77 (0.62, 0.96) | 0.71 (0.55, 0.91) |
| Sensitivity analysis 6 | 808/26103 | 1.00 (reference) | 0.75 (0.61, 0.93) | 0.70 (0.56, 0.87) | 0.61 (0.49, 0.76) |
| Sensitivity analysis 7 | 157/26103 | 1.00 (reference) | 0.83 (0.53, 1.28) | 0.56 (0.34, 0.91) | 0.49 (0.30, 0.79) |
| **Soy isoflavones** | | | | | |
| Main analysis | 900/27003 | 1.00 (reference) | 0.83 (0.68, 1.01) | 0.74 (0.60, 0.90) | 0.70 (0.58, 0.86) |
| Sensitivity analysis 1 | 802/22074 | 1.00 (reference) | 0.77 (0.63, 0.95) | 0.72 (0.58, 0.89) | 0.69 (0.56, 0.85) |
| Sensitivity analysis 2 | 856/24827 | 1.00 (reference) | 0.81 (0.66, 0.99) | 0.74 (0.60, 0.91) | 0.66 (0.54, 0.81) |
| Sensitivity analysis 3 | 900/27003 | 1.00 (reference) | 0.82 (0.68, 1.00) | 0.74 (0.60, 0.90) | 0.70 (0.57, 0.85) |
| Sensitivity analysis 4 | 2449/27003 | 1.00 (reference) | 0.94 (0.83, 1.06) | 0.78 (0.69, 0.89) | 0.73 (0.65, 0.83) |
| Sensitivity analysis 5 | 900/27003 | 1.00 (reference) | 0.87 (0.71, 1.06) | 0.85 (0.65, 1.11) | 0.80 (0.64, 0.97) |
| Sensitivity analysis 6 | 808/26103 | 1.00 (reference) | 0.82 (0.67, 1.00) | 0.72 (0.58, 0.89) | 0.70 (0.56, 0.86) |
| Sensitivity analysis 7 | 157/26103 | 1.00 (reference) | 0.91 (0.58, 1.40) | 0.62 (0.38, 0.99) | 0.54 (0.34, 0.87) |
| **Daidzein** | | | | | |
| Main analysis | 900/27003 | 1.00 (reference) | 0.77 (0.63, 0.94) | 0.62 (0.49, 0.78) | 0.58 (0.47, 0.72) |
| Sensitivity analysis 1 | 802/22074 | 1.00 (reference) | 0.81 (0.66, 1.00) | 0.60 (0.47, 0.75) | 0.56 (0.45, 0.70) |
| Sensitivity analysis 2 | 856/24827 | 1.00 (reference) | 0.81 (0.67, 0.99) | 0.58 (0.46, 0.72) | 0.54 (0.43, 0.67) |
| Sensitivity analysis 3 | 900/27003 | 1.00 (reference) | 0.80 (0.66, 0.97) | 0.59 (0.47, 0.73) | 0.55 (0.45, 0.68) |
| Sensitivity analysis 4 | 2449/27003 | 1.00 (reference) | 0.88 (0.78, 0.99) | 0.65 (0.57, 0.75) | 0.62 (0.54, 0.70) |
| Sensitivity analysis 5 | 900/27003 | 1.00 (reference) | 0.83 (0.68, 1.01) | 0.62 (0.49, 0.78) | 0.60 (0.47, 0.77) |
| Sensitivity analysis 6 | 808/26103 | 1.00 (reference) | 0.82 (0.67, 1.00) | 0.57 (0.45, 0.72) | 0.55 (0.44, 0.69) |
| Sensitivity analysis 7 | 157/26103 | 1.00 (reference) | 0.62 (0.40, 0.98) | 0.51 (0.31, 0.84) | 0.38 (0.23, 0.63) |
| **Glycitein** | | | | | |
| Main analysis | 900/27003 | 1.00 (reference) | 0.77 (0.64, 0.91) | 0.49 (0.39, 0.60) | 0.45 (0.36, 0.57) |
| Sensitivity analysis 1 | 802/22074 | 1.00 (reference) | 0.74 (0.59, 0.91) | 0.61 (0.48, 0.77) | 0.58 (0.46, 0.73) |
| Sensitivity analysis 2 | 856/24827 | 1.00 (reference) | 0.79 (0.64, 0.96) | 0.60 (0.48, 0.76) | 0.58 (0.47, 0.72) |
| Sensitivity analysis 3 | 900/27003 | 1.00 (reference) | 0.75 (0.62, 0.92) | 0.61 (0.48, 0.76) | 0.57 (0.46, 0.70) |
| Sensitivity analysis 4 | 2449/27003 | 1.00 (reference) | 0.85 (0.75, 0.96) | 0.63 (0.54, 0.72) | 0.59 (0.51, 0.67) |
| Sensitivity analysis 5 | 900/27003 | 1.00 (reference) | 0.80 (0.65, 0.97) | 0.65 (0.52, 0.82) | 0.64 (0.51, 0.81) |

| | | | | | |
|---|---|---|---|---|---|
| Sensitivity analysis 6 | 808/26103 | 1.00 (reference) | 0.79 (0.64, 0.97) | 0.60 (0.47, 0.76) | 0.56 (0.45, 0.71) |
| Sensitivity analysis 7 | 157/26103 | 1.00 (reference) | 0.39 (0.24, 0.65) | 0.58 (0.35, 0.94) | 0.41 (0.24, 0.67) |
| **Genistein** | | | | | |
| Main analysis | 900/27003 | 1.00 (reference) | 0.88 (0.72, 1.06) | 0.79 (0.65, 0.97) | 0.66 (0.55, 0.83) |
| Sensitivity analysis 1 | 802/22074 | 1.00 (reference) | 0.82 (0.67, 1.01) | 0.78 (0.63, 0.97) | 0.66 (0.53, 0.82) |
| Sensitivity analysis 2 | 856/24827 | 1.00 (reference) | 0.86 (0.71, 1.05) | 0.79 (0.64, 0.97) | 0.63 (0.51, 0.78) |
| Sensitivity analysis 3 | 900/27003 | 1.00 (reference) | 0.87 (0.72, 1.01) | 0.79 (0.65, 0.97) | 0.67 (0.55, 0.82) |
| Sensitivity analysis 4 | 2449/27003 | 1.00 (reference) | 0.88 (0.78, 0.99) | 0.80 (0.70, 0.91) | 0.70 (0.61, 0.79) |
| Sensitivity analysis 5 | 900/27003 | 1.00 (reference) | 0.92 (0.75, 1.12) | 0.85 (0.68, 1.06) | 0.77 (0.60, 0.98) |
| Sensitivity analysis 6 | 808/26103 | 1.00 (reference) | 0.87 (0.71, 1.07) | 0.76 (0.61, 0.94) | 0.68 (0.55, 0.84) |
| Sensitivity analysis 7 | 157/26103 | 1.00 (reference) | 1.08 (0.69, 1.67) | 0.81 (0.51, 1.30) | 0.53 (0.32, 0.86) |

CKD, chronic kidney disease; OR, odds ratio; SD, standard deviation; CI, confidence interval.

The lowest quartile (Q1) of each variable was set as the reference group. Ors and 95% CIs were estimated using logistic regression models. *P* for trend was calculated by assigning the median value of each quartile to the respective group and treating it as a continuous variable.

The model was adjusted for age, sex, ethnicity, BMI, hypertension, diabetes, smoking status, drinking status, annual family income, education, physical activity, energy intake, and dietary pattern (derived without beans and bean products).

[1] Sensitivity analysis 1: Exclude participants under the age of 40；

[2] Sensitivity analysis 2: Exclude participants with changes in dietary habits (during the previous year);

[3] Sensitivity analysis 3: Include hyperuricemia as a covariate in the model for adjustment；

[4] Sensitivity analysis 4: Using the K/DOQI guidelines developed by the National Kidney Foundation as the diagnostic basis for CKD;

[5] Sensitivity analysis 5: Include total protein, sodium, and red meat intake as covariates in the model for adjustment;

[6] Sensitivity analysis 6: Using urine protein positivity as the outcome, excluding participants with isolated reduced eGFR;

[7] Sensitivity analysis 7: Using reduced eGFR as the outcome, excluding participants with isolated urine protein positivity.

**Supplementary Table 5 Comparison of baseline characteristics between excluded and included groups**

| **Variables** | **Excluded group (n=9373)** | **Included group (n=27003)** | **Z/χ2** | ***P*-value** |
|---|---|---|---|---|
| **Age, years, n (%)** | | | 18.839 | <0.001 |
| <50 | 2695 (31.87) | 9296 (34.43) | | |
| ≥50 | 5762 (68.13) | 17707 (65.57) | | |
| **Sex, n (%)** | | | 6.327 | 0.012 |
| Men | 2855 (33.59) | 9472 (35.08) | | |
| Women | 5645 (66.41) | 17531 (64.92) | | |
| **Ethnicity, n (%)** | | | 254.00 | <0.001 |
| Han | 3722 (44.07) | 14581 (54.00) | | |
| Other | 4724 (55.93) | 12422 (46.00) | | |
| **Education, n (%)** | | | 266.479 | <0.001 |
| Middle school and below | 5925 (69.79) | 16188 (59.95) | | |
| High school | 1297 (15.28) | 5452 (20.19) | | |
| College and above | 1268 (14.94) | 5363 (19.86) | | |
| **Annual family income, RMB, n (%)** | | | 464.965 | <0.001 |
| <50,000 | 4700 (55.87) | 11516 (42.66) | | |
| 50,000-100,000 | 2758 (32.78) | 10983 (40.68) | | |
| >100,000 | 955 (11.35) | 4498 (16.66) | | |
| **Current smoker, n (%)** | 1711 (20.14) | 5862 (21.71) | 9.520 | 0.002 |
| **Current drinker, n (%)** | 1555 (18.30) | 6073 (22.49) | 67.091 | <0.001 |
| **Physical activity, MET-h/d ,n (%)** | | | 229.445 | <0.001 |
| ≤50 | 3334 (35.57) | 7379 (27.33) | | |
| 51-100 | 2560 (27.31) | 8405 (31.13) | | |
| 101-150 | 1451 (15.48) | 4816 (17.83) | | |
| ＞150 | 2028 (21.64) | 6403 (23.71) | | |
| **BMI, kg/m$^2$, n (%)** | | | 13.112 | <0.001 |

| | | | | |
|---|---|---|---|---|
| <24 | 3259 (39.11) | 9969 (36.92) | | |
| ≥24 | 5073 (60.89) | 17034 (63.08) | | |
| **Waist, n (%)** | | | 83.292 | <0.001 |
| Normal | 4531 (53.31) | 12862 (47.63) | | |
| Obesity | 3969 (46.69) | 14141 (52.37) | | |
| **SBP, mmHg, median, (IQR)** | 132.00 (119.67, 147.33) | 131.34 (119.67, 145.67) | 2.821 | 0.005 |
| **DBP, mmHg, median, (IQR)** | 79.67 (72.33, 87.33) | 79.34 (72.00, 87.00) | 1.893 | 0.058 |
| **FBG, mmol/L, median, (IQR)** | 5.24 (4.80, 5.88) | 5.33 (4.91, 5.90) | -9.843 | <0.001 |
| **SUA, µmol/L, median, (IQR)** | 295.07 (243.70, 358.40) | 302.00 (252.70, 364.20) | -6.972 | <0.001 |
| **Hypertension, n (%)** | 3665 (39.10) | 11087 (41.06) | 11.050 | 0.001 |
| **Diabetes, n (%)** | 1112 (11.86) | 3146 (11.65) | 0.306 | 0.580 |
| **Hyperuricemia, n (%)** | 718 (10.64) | 3120 (11.55) | 4.512 | 0.034 |
| **Energy intake, kcal/d, median, (IQR)** | 1183.93 (799.20, 1613.61) | 1371.09 (1045.71, 1796.07) | -29.575 | <0.001 |
| **Carbohydrate intake, g/d, median, (IQR)** | 185.30 (131.60, 245.09) | 209.81 (163.25, 269.46) | -26.960 | <0.001 |
| **Protein intake, g/d, median, (IQR)** | 47.23 (32.41, 64.69) | 55.28 (42.00, 73.55) | -30.724 | <0.001 |
| **Fat intake, g/d, median, (IQR)** | 26.70 (16.94, 39.83) | 32.92 (22.89, 46.88) | -29.496 | <0.001 |
| **Meat intake, g/d, median, (IQR)** | 26.34 (9.84, 57.12) | 42.84 (17.16, 64.92) | -28.277 | <0.001 |
| **Fish intake, g/d, median, (IQR)** | 17.22 (7.15, 25.83) | 20.45 (9.84, 31.69) | -18.287 | <0.001 |
| **Vegetable intake, g/d, median, (IQR)** | 166.54 (102.90, 266.12) | 193.14 (123.57, 291.65) | -16.988 | <0.001 |
| **Fruit intake, g/d, median, (IQR)** | 94.29 (32.80, 198.69) | 115.62 (49.86, 224.90) | -12.291 | <0.001 |
| **Total beans and bean products residual-adjusted intake, g/d, median, (IQR)** | 77.50 (54.43, 108.67) | 68.48 (44.77, 100.92) | 19.485 | <0.001 |
| **Beans residual-adjusted intake [1], g/d, median, (IQR)** | 19.39 (13.12, 27.80) | 17.52 (11.70, 25.30) | 16.455 | <0.001 |
| **Bean products residual-adjusted intake [2], g/d, median, (IQR)** | 55.47 (36.95, 81.77) | 48.70 (28.12, 76.69) | 18.232 | <0.001 |
| **Soy isoflavones residual-adjusted intake [3], mg/d, median, (IQR)** | 15.24 (10.44, 22.39) | 13.93 (8.68, 21.33) | 14.996 | <0.001 |

| | | | | |
|---|---|---|---|---|
| Daidzein intake | 2.70 (1.82, 3.78) | 2.32 (1.41, 3.45) | 21.852 | <0.001 |
| Glycitein intake | 2.03 (1.18, 2.92) | 1.61 (0.77, 2.68) | 23.134 | <0.001 |
| Genistein intake | 2.40 (1.67, 3.40) | 2.20 (1.43, 3.24) | 15.427 | <0.001 |

CKD, chronic kidney disease; BMI, body mass index; MET, metabolic equivalent; IQR, interquartile range; SBP, systolic blood pressure; DBP, diastolic blood pressure; FBG, fasting blood glucose; SUA, serum uric acid.

Since none of the continuous variables followed a normal distribution, the Wilcoxon rank-sum test was used to compare differences between groups, and variables were described using medians (25th percentile, 75th percentile). For categorical variables, chi-square tests were employed to calculate *P*-values, and distributions were presented as counts (percentages). Intake of total beans and bean products, beans, and bean products was adjusted for energy intake using the residual method. The resulting values represent relative intake levels independent of total energy.

[1] Beans include: soybeans, mung beans, various snap beans, bean sprouts and other beans;

[2] Beans products include: soy milk, soybean milk, tofu pudding, tofu, dried tofu sticks and dried tofu;

[3] The free aglycones of soy isoflavones are primarily classified into three types: daidzein, glycitein and genistein.
